# Assessment of Plant Sterols in the Diet of Adult Polish Population with the Use of a Newly Developed Database

**DOI:** 10.3390/nu13082722

**Published:** 2021-08-07

**Authors:** Anna Maria Witkowska, Anna Waśkiewicz, Małgorzata Elżbieta Zujko, Iwona Mirończuk-Chodakowska, Alicja Cicha-Mikołajczyk, Wojciech Drygas

**Affiliations:** 1 Department of Food Biotechnology, Faculty of Health Sciences, Medical University of Bialystok, Szpitalna 37, 15-295 Bialystok, Poland; malgorzata.zujko@umb.edu.pl (M.E.Z.); iwona.mironczuk-chodakowska@umb.edu.pl (I.M.-C.); 2 Department of Epidemiology, Cardiovascular Disease Prevention and Health Promotion, National Institute of Cardiology, Alpejska 42, 04-628 Warsaw, Poland; awaskiewicz@ikard.pl (A.W.); acicha@ikard.pl (A.C.-M.); wdrygas@ikard.pl (W.D.); 3 Department of Social and Preventive Medicine, Faculty of Health Sciences, Medical University of Lodz, Hallera 1, 90-001 Lodz, Poland

**Keywords:** plant sterols, database, Polish population

## Abstract

Plant sterols are compounds with multiple biological functions, mainly cholesterol-reducing. There are no comprehensive databases on plant sterols, which makes it difficult to estimate their intake in the Polish population. This work attempted to use international food databases, additionally supplemented by scientific data from the literature, to create a database of plant sterols, which would cover various kinds of foods and dishes consumed in Poland. The aim was to assess the size and sources of dietary plant sterols in the adult population of Poland. The literature search was conducted using PubMed, Web of Science, Scopus, and Google Scholar to identify possible sources of published food composition data for plant sterols. The study group consisted of 5690 participants of the WOBASZ II survey. We identified 361 dietary sources of plant sterols based on the consumption of foods and dishes reported by participants. Cereals and fats provided 61% of the total plant sterols, and together with vegetables and fruits, this totaled 80%. The median intake of plant sterols in the Polish population was 255.96 mg/day, and for men and women 291.76 and 230.61 mg/day, respectively. Canola oil provided the most plant sterols at 16.92%, followed by white bread at 16.65% and soft margarine at 8.33%. The study found that plant sterol intake in Poland is comparable to other populations, and women’s diets are more dense in plant sterols. Due to the lack of literature sources on plant sterol content in some foods, future studies should expand and complete the databases on plant sterol content in foods.

## 1. Introduction

Plant sterols are bioactive phytocompounds with a molecular structure similar to cholesterol [1]. The absorption of dietary cholesterol from diets rich in phytosterols is reduced by various mechanisms, mainly associated with the displacement of cholesterol from lipid micelles [2]. To date, more than 250 phytosterols have been identified, which include plant sterols and their saturated forms, stanols [3,4]. In various food sources, β-sitosterol is predominant and accounts for approximately 80% of the phytosterol intake in the diet [5]. Clinical evidence shows that phytosterols have a moderate LDL- and triglyceride-lowering effect [6,7]. Phytosterols are also considered moderately active antioxidants [8] and have immunomodulatory properties [9]. Sitosterol may suppress obesity-related chronic inflammation by reducing circulating interleukin-6 and TNF-α [10]. A growing body of evidence suggests that phytosterols may be an alternative and/or complementary therapy for patients with obesity and diabetes [3]. The consumption of naturally occurring plant sterols has been found to be associated with a lower risk of first myocardial infarction in men [11]. In addition, high doses of plant sterols in the diet, especially β-sitosterol, have been found to prevent the development of cancer [12,13].

Food sources with the highest plant sterol content include vegetable oils, mainly corn oil (746 mg/100 g), and sesame seeds (714 mg/100 g) [14]. A good source of phytosterols is nuts, which provide 30–220 mg/100 g of phytosterols, and cereals that contain phytosterols in the amount of 35–198 mg/100 g [15]. Vegetables contain smaller amounts of phytosterols, with 4–40 mg/100 g, and fruits contain 4–24 mg/100 g [15]. Consumption studies have shown that due to the frequency and volume of consumption, the suppliers of plant sterols are mainly bread, cereals, fats, and vegetables [3,5]. As studies show, population intakes of plant sterols are variable [5,11,16,17,18,19,20,21].

There is a need to develop databases of biologically active compounds to calculate population intakes [22]. Unlike the various databases on food composition, there are no comprehensive databases on plant sterols, which makes it difficult to estimate the intake of plant sterols in populations, as well as their further calculations in epidemiological studies. Earlier population-based studies used different databases prepared for individual studies with different methodologies [5,11,16,17,18,19,20,21]. Some studies used plant sterol databases [16,18,20], but others prepared individual databases based on experimental data [5,11,17,19,21]. There is currently no evaluation of plant sterols at the Polish population level, but an attempt has been made in a pilot study on a sample of students [23].

This work attempted to use international food databases, additionally supplemented by scientific data from the literature, to create a database of plant sterols, which would cover various kinds of foods and dishes consumed in Poland. The aim was to assess the size and sources of dietary plant sterols in the adult population of Poland.

## 2. Materials and Methods

### 2.1. Plant Sterol Database and Calculation of Dietary Intake

Since there is no plant sterol database in Poland, its establishment for the purpose of this study was based on international databases, which were published in English and are publicly available [14,24]. A literature review was conducted to search for reliable data sources that would supplement the data taken from international databases. The literature search was conducted using PubMed, Web of Science, Scopus, and Google Scholar to identify possible sources of published food composition data for plant sterols. The search terms included phytosterols, plant sterols, β-sitosterol, campesterol, and stigmasterol combined with food, cereals, vegetables, fruit, berries, nuts, seeds, legumes, beverages, coffee, tea, wine, soda, chocolate, pastry, and cookies.

The plan was to select data sources that were as complete as possible in terms of individual plant sterols (β-sitosterol, campesterol, and stigmasterol). For the total plant sterol content, the full data reported by databases or scientific sources were used or, in the absence of relevant data, the available data for plant sterol content were aggregated. The quality of the data was assessed according to the procedure described by Rand et al. [25], which takes into account the analytical method used, the number of samples, the sample handling procedures, the sampling plan for the selection of foods, and the analytical and quality assurance. The currently available techniques for sterol analysis are gas chromatography (GC), high-pressure liquid chromatography (HPLC), and supercritical fluid chromatography (SFC). GC/FID (flame-ionization detection) or GC/MS (mass spectrometry) can be considered the methods of choice for the determination of phytosterols in foods and diets [26]. For most of the studies, all of the quality criteria were met. For some food products, the number of studies was limited to only one publication; although they did not meet all quality criteria, they were included in the developed database due to lack of other publication sources. Finally, data from 13 data sources were included in the database, with 11 studies meeting the Rand criteria and 2 not meeting these criteria.

In this study, data for fats and oils were extracted from the British database of Food Composition [24], the USDA Database [14], and Normen et al. [27]. Data on plant sterols in cereals were extracted from the British database of Food Composition [24] and Normen et al. [28]. Most of the data for vegetables and potatoes were taken from Normen et al. [29]. Data gaps in the vegetables group were filled in from the publications by Han et al. [30], Piironen et al. [31], Ryan et al. [32], the British database of Food Composition [24], and the USDA Nutrient Database [14]. The plant sterol contents in fruits and berries were compiled from the USDA Database [14], Piironen et al. [31], Normen et al. [29], and Han et al. [30]. The plant sterol contents in nuts and seeds were taken from the USDA Database [14], the British database of Food Composition [24], and Normen et al. [27]. The plant sterols for legumes were compiled from Li et al. [33], Han et al. [30], the USDA Database [14], Ryan et al. [32], and Yamaya et al. [34]. Data for fruit and vegetable juices, sodas, tea, and beer were taken from Decloedt et al. [35]. Data for the plant sterols in wines were taken from Ruggiero et al. [36]. The plant sterol content in the sterolic fraction of coffee was taken from Čížková et al. [37] and recalculated per 100 g of coffee. For pastry and cookies, data were extracted from the British database of Food Composition [24], the USDA Database [14], and Piironen et al. [31]. For chocolate and chocolate candies, data were compiled from Normen et al. [27]. Data on plant sterols in foods are available in Supplementary Table S1.

For the dishes, the individual ingredients were extracted according to recipes of the National Institute of Food and Nutrition of Poland, taking into account the yield factors of the dishes. Data on plant sterols in dishes are available in Supplementary Table S2.

Finally, foods were grouped into 10 categories: cereals (flour, bread, breakfast cereals, bran, groats, and pasta), fruit (processed and non-processed), vegetables (processed and non-processed), potatoes, legumes, fats and oils (oils, margarine, and mayonnaise), coffee (instant and infusion), cookies and cakes, chocolate (chocolate and chocolate candies and bars), and other foods (tea, beer, wine, sodas, mustard, nuts, and seeds). Foods enriched with phytosterols were not included in these calculations because not all manufacturers were willing to disclose their formulations regarding individual phytosterols.

**Figure 1:**
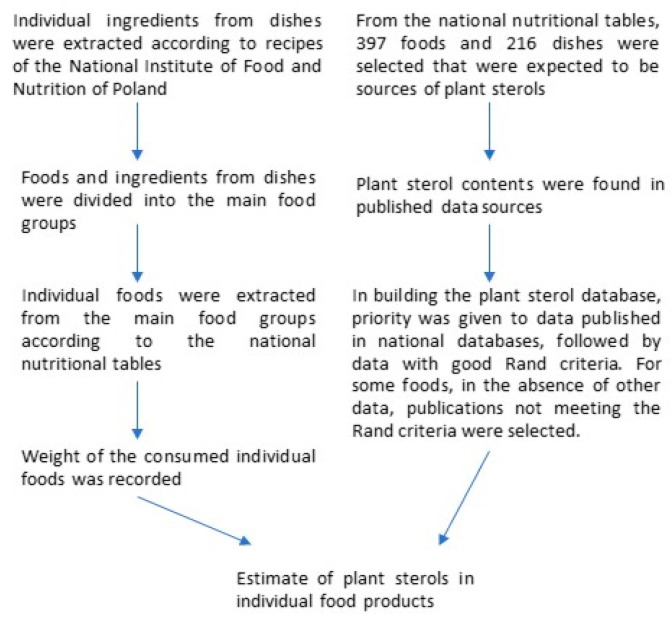
The process used to estimate plant sterol intake.

The process used to estimate plant sterols in foods is given in Figure 1.

### 2.2. Study Group and Data Collection

**Figure 2:**
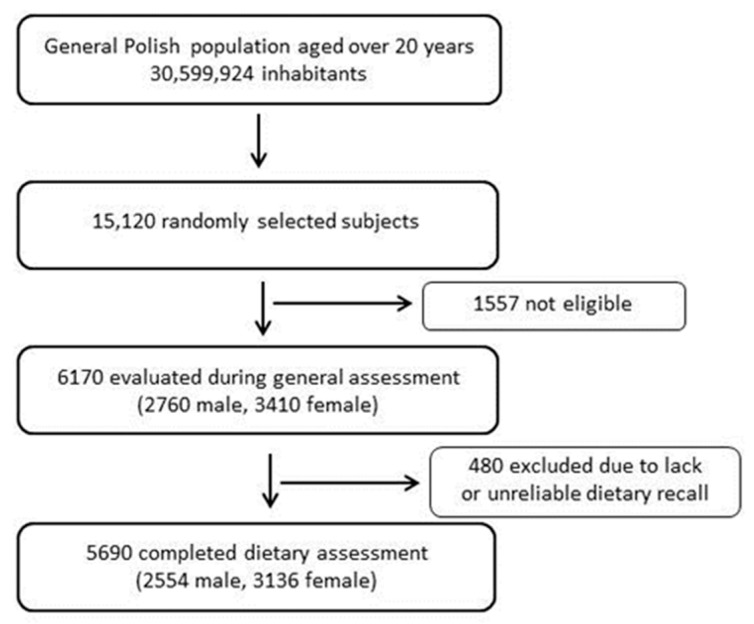
Flow chart of the study participants.

The study group consisted of 5690 participants (2554 men and 3136 women) of the National Multicenter Health Survey II (the Polish acronym is WOBASZ II). WOBASZ II is a cross-sectional study representative of the Polish adult population aged 20 years and over, which was carried out by the National Institute of Cardiology (formerly the Institute of Cardiology), Warsaw, Poland, in the years 2013–2014, in collaboration with five national medical universities. The design and methods of the WOBASZ II survey have been described in detail elsewhere [38]. Daily food consumption data were collected by trained interviewers using a single 24-h dietary recall method. The overall evaluation included a sample of 6170 participants, 480 of whom were excluded due to missing or unreliable dietary recalls. A flowchart of the participants is shown in Figure 2. The WOBASZ II study was approved by the Bioethics Committee of the National Institute of Cardiology (no. 1344), as was the current study (no. 1837). Written informed consent was obtained from all participants.

Data on the demographic status, diseases, leisure-time physical activity, tobacco use, community size, marital status, and education level of the participants were collected using a standardized questionnaire developed for the WOBASZ II survey. Height and weight measurements were taken by personnel trained in standard procedures. Body mass index (BMI) was calculated from body weight in kilograms divided by the square of the height in meters. Blood pressure (BP) was measured three times on the right arm after 5 min of rest in a sitting position at 1 min intervals, and final BP was reported as the mean of the second and third measurements. The general characteristics of the study group are shown in Table 1.

The present study identified 361 dietary sources of plant sterols based on the consumption of foods and dishes reported by participants in the WOBASZ II survey. A small proportion of subjects who consumed phytosterol-enriched products was found (Table 1). Plant sterol daily intake was determined by multiplying the daily consumption of individual food items by the respective total plant sterols, such as the β-sitosterol, campesterol, and stigmasterol contents, in these food items and then summed up.

### 2.3. Data Analysis

Total phytosterol intake, including β-sitosterol, campesterol, and stigmasterol, was calculated by multiplying the daily consumption of individual food items by the respective phytosterol contents in these products. Additionally, the contribution of individual groups of food products and their ingredients to the consumption of different phytosterols was studied. Descriptive statistics were applied to describe the continuous variables (means and standard deviations, as well as median and interquartile range), and the percentages of the respective values were used for categorized variables. The contributions of food categories and individual food items to the intake of particular total and individual phytosterols are presented as percentages. To investigate the differences between men and women, a non-parametric Wilcoxon test or Chi-square test was used, respectively, for quantitative and qualitative variables. The level of significance was considered *p* < 0.05. Data analyses were processed using Statistical Analysis System (SAS; version 9.4, SAS Institute Inc., Cary, NC, USA).

## 3. Results

This study identified the top 10 food categories that provided plant sterols for the Polish population, which were cereals, vegetable fats and oils, vegetables, fruits, coffee, cookies and cakes, chocolate products, potatoes, and legumes. The other food products providing lower amounts of plant sterols were classified into the category of “other food products”. Among all of these categories, cereals and fats provided 61% of the total plant sterols, and together with vegetables and fruits, this totaled 80%. Median total plant sterol intake in this study was 255.96 mg/day, and divided by men and women was 291.76 and 230.61 mg/day, respectively (Table 2). Considering individual foods (mg/day), canola oil provided the most plant sterols at 16.92%, followed by white bread at 16.65% and soft margarine at 8.33%. Among vegetables and fruits, there was no single significant source of plant sterols, but raw fruits and vegetables provided the predominant amounts of plant sterols (9.78% and 7.27%, respectively). This pattern of plant sterol sources was reflected in men, while among women, the main contributor was canola oil, followed by white bread, raw fruits, raw vegetables and soft margarine. Gender differences were found for most sources of plant sterol intake.

**Figure 3:**
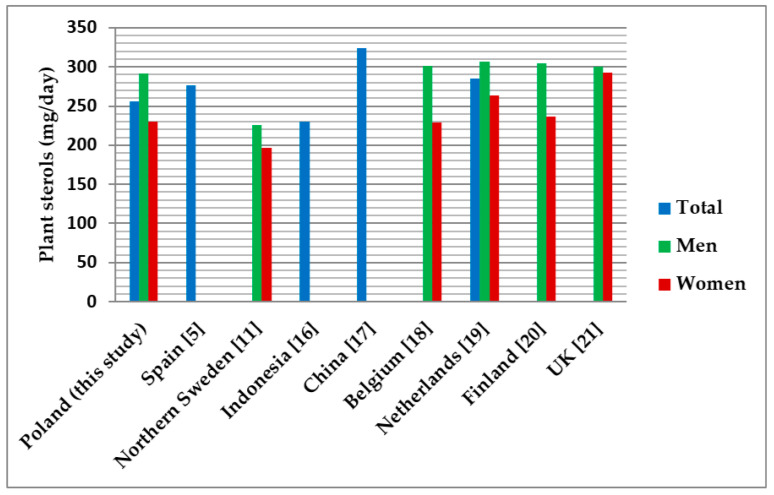
Intake of plant sterols in the Polish population and in other countries.

Figure 3 shows the intake of plant sterols in the Polish population (total, men, women) compared to other populations. With a plant sterol intake of 255.96 mg/day, the data for Poles are within the range for other populations.

Table 3, Table 4 and Table 5 show the contribution of food categories to the consumption of individual plant sterols such as β-sitosterol, campesterol, and stigmasterol. The median β-sitosterol consumption was 160.85 mg/day, while the intake of campesterol and stigmasterol was 47.45 mg/day and 22.10 mg/day, respectively.

The main food categories providing β-sitosterol were cereals (29.19%), fats (28.86%), fruits (14.20%), and vegetables (8.70%), with a total share of 80.95% of the β-sitosterol supply (Table 3). Among the food products, β-sitosterol was supplied by canola oil (15.88%), followed by wheat bread (14.88%) and soft margarine (9.02%). Women had a lower β-sitosterol intake compared to men at 146.28 mg/day vs. 180.84 mg/day, respectively.

The main sources of campesterol were fats (44.95%) and cereal products (31.81%), which together accounted for 76.76% of the campesterol intake (Table 4). For individual products, campesterol was supplied by canola oil (33.43%), white bread (16.30%), and soft margarines (8.11%). Men consumed more campesterol compared to women (56.71 vs. 40.88 mg/day, respectively).

As for stigmasterol, its main sources were the following product groups: coffee (25.10%), vegetables (23.22%), fats (16.85%), and cereal products (12.93%). The foods supplying the highest amounts of stigmasterol included coffee (as a food product; 25.10%), soft margarine (11.82%), and white bread (6.48%). The median intake of stigmasterol was higher in men at 23.49 mg/day compared to women at 21.11 mg/day.

On a per milligram basis, men consumed more total and individual plant sterols (Table 6). However, per 1000 kcal, significantly more plant sterols as total and individual sterols were consumed by women (*p* < 0.0001), except for campesterol, for which the difference was not statistically significant.

## 4. Discussion

This is the first report on dietary plant sterol intake and its dietary sources in the Polish population. Due to the lack of plant sterols in Polish food composition tables, the database used for this study included international databases available in English supplemented with data from research papers on plant sterol contents in food products. In our study, the consumption of plant sterols from enriched food products was not taken into consideration, since the percentage of consumers of phytosterol-enriched products was low (2%). In comparison, it has been estimated that regular consumers of products with added plant sterols represent approximately 10–15% of the EU population [39].

Typical contemporary Western diets provide much lower amounts of phytosterols [40] than estimated for distant human ancestors, whose diet provided 1 g/day of phytosterols [41]. The dietary phytosterol intake in population studies is usually between 200 and 400 mg/day [21,42], even in those populations with more beneficial dietary habits [43], and this amount is too low to show significant LDL cholesterol-lowering effects demonstrated for 1 g of phytosterols [44]. Contrary to this, the PREDIMED study found that even small amounts of plant sterols from natural foods may exert a cholesterol-lowering effect [45]. A recent meta-analysis of 124 clinical studies demonstrated that a phytosterol intake between 0.6 and 3.3 g/day is associated with a gradual decrease in the concentration of LDL-cholesterol from 6% to 12% [46]. Scientific evidence indicates that even moderate doses of phytosterols delivered via a normal diet can provide a protective effect on the lipid profile by reducing cholesterol absorption [47,48], but a lipid-lowering effect may depend on the inter-individual variation in response to phytosterols [49].

The daily intake of total plant sterols in our study (255.96 mg/day) is similar to that of the Spanish population, where it was estimated to be 276 mg, with the largest contribution of beta-sitosterol (79.7%) [5]. In different populations, plant sterol intake ranged from 230 to 324 mg/day. Among other things, these differences may be due to the dietary habits of different populations or the availability of different food products on the market. Some differences may also be due to the food intake methodology. Some studies were based on a 24-h interview or dietary records, and others on a frequency of intake. Our results confirm earlier findings that β-sitosterol is the most important contributor (67.8%) to the intake of total dietary plant sterols. Regarding gender differences in plant sterol intake, in our study, the intake was 291.76 mg/day for men and 230.61 mg/day for women. These results are similar to most other populations where gender differences in plant sterol intake were observed among men and women [20]. These differences may be due to differences in food intake between the two sexes. Women tend to consume smaller portions of foods, which translates into fewer ingredients including plant sterols.

As per our study, the consumption pattern of total plant sterols from major food groups such as cereal products, vegetable oils and fats, vegetables, and fruits is similar to the intervention group in the PREDIMED study and to the U.K. population [21,45]. Of these, cereal products and oils provided nearly 61% of plant sterols, and when combined with vegetables and fruits, nearly 80%. However, unlike the PREDIMED study, where legumes were the fifth contributor to total plant sterols, in our study, the additional sources of plant sterols included coffee, cookies and cakes, chocolate products, and potatoes, while legumes were only ninth in providing plant sterols. Together, these minor sources of plant sterols accounted for 16.68% of plant sterol intake. The other sources of plant sterols accounted for 3.55%; these included, among others, nuts and seeds, which are normally a good source of plant sterols, but because of their low intake [50], they were not a significant source of plant sterols for the Polish population. The PREDIMED intervention study indicated an important role for the Mediterranean diet, in combination with nuts, in providing plant sterols in the diet and providing a cholesterol-lowering effect [45]. Considering this, Poles should be encouraged to increase their nut consumption and improve their dietary habits, which are far from the recommended for the prevention of cardiovascular diseases [51,52]. Regarding individual dietary sources of total plant sterols, canola oil and white bread predominated, followed by soft margarine. Similar to a Chinese study, canola oil was the main provider of plant sterols among vegetable fats and oils [53].

As in the study of EPIC-Norfolk population [21], women in the WOBASZ survey had a higher plant sterol density than men. Interestingly, when converted per 1000 kcal, the total plant sterol content did not differ from the values obtained in the EPIC-Norfolk study. For men and women in our study, the amount of plant sterols was 141.0 mg and 154.2 mg, respectively, and in the EPIC-Norfolk study, for men it was 137.33 mg and for women it was 152.4 mg/day.

### Limitations

Some plant sterol values in this study may have been underestimated because only three major sterols (sitosterol, campesterol, and stigmasterol) are typically included in the totals, despite the contribution of other sterols. Although the compiled database facilitated the calculation of plant sterols, there are some shortcomings due to the lack of data for individual plant sterols. This is mainly due to the fact that the literature data do not provide information on the content of plant sterols in certain food products. For some foods, the values of plant sterols (total and individual) were not found, e.g., no studies were found for chard. No data were found for campesterol or stigmasterol in radishes, wines, and mushrooms. For foods such as chives, blueberries, cherries, pears, raspberries, blackcurrants, walnuts, and pumpkin seeds, no value was found for stigmasterol. Therefore, the values obtained for the sum of individual plant sterols could be lower than the total plant sterol content. Moreover, there are no specific data on the composition of plant sterols in enriched margarine, which is related, among other things, to proprietary manufacturing technologies. In addition, since a small percentage of study participants consumed phytosterol-enriched margarine, they were not included in the calculation of dietary plant sterols.

Furthermore, a limitation of the study is the inclusion in the plant sterol database of results from several less rigorous literature sources than those given in the Rand criteria.

This study used single 24-h recall as a tool to measure food intake, which is an appropriate method for large-scale studies. However, 24-h recall does not account for variability in food intake and may not describe a typical diet.

## 5. Conclusions

This is the first study to evaluate the intake of plant sterols in the Polish population. Since no plant sterols are listed in Polish food composition tables, a database was developed using published data sources. The study found that plant sterol intake in Poland is 255.96 mg/day, which is comparable to other populations, despite different methodologies of nutritional assessment and slightly different databases. The main dietary sources of plant sterols in this study were cereals, fats, vegetables, and fruits, which is consistent with data for other populations. This study found that women’s diets are more dense in plant sterols, which is in agreement with other studies. Due to the lack of literature sources on plant sterol content in some foods, future studies should expand and complete the databases on plant sterol content in foods.

## Figures and Tables

**Table 1:** General description of the studied population.

Trait	Men and Women N = 5690	Men N = 2554	Women N = 3136	*p* *
Age (year), mean ± SD median (IQR)	49.58 ± 16.43 50.00 (36.00–62.00)	48.79 ± 16.27 49.00 (35.00–61.00)	50.23 ± 16.54 51.00 (37.00–62.00)	0.0023
BMI (kg/m^2^), mean ± SD median (IQR)	27.17 ± 5.19 26.63 (23.54–30.15)	27.42 ± 4.55 27.07 (24.34–30.02)	26.96 ± 5.65 26.12 (22.87–30.39)	<0.0001
Systolic BP (mmHg), mean ± SD median (IQR)	130.67 ± 19.34 127.5 (117.5–141.0)	134.44 ± 18.19 131.5 (122.0–144.5)	127.6 ± 19.71 124.0 (113.5–138.0)	<0.0001
Diastolic BP (mmHg), mean ± SD median (IQR)	80.23 ± 10.81 80.0 (72.5–87.0)	81.51 ± 10.91 81.0 (74.0–88.0)	79.19 ± 10.62 78.5 (72.0–85.5)	<0.0001
Fasting glucose (mmol/L), mean ± SD median (IQR)	5.50 ± 1.46 5.21 (4.84–5.72)	5.65 ± 1.6 5.35 (4.96–5.84)	5.38 ± 1.32 5.12 (4.77–5.58)	<0.0001
Total cholesterol (mmol/L), mean ± SD median (IQR)	5.20 ± 1.27 5.14 (4.38–5.93)	5.21 ± 1.33 5.15 (4.36–5.97)	5.19 ± 1.22 5.14 (4.41–5.90)	0.7223
LDL-cholesterol (mmol/L), mean ± SD median (IQR)	3.15 ± 1.03 3.07 (2.42–3.78)	3.19 ± 1.04 3.15 (2.46–3.86)	3.11 ± 1.02 3.01 (2.39–3.72)	0.0002
Diseases (%)				
Hypertension ^1^	45.22	49.56	41.69	<0.0001
Hypercholesterolemia ^2^	67.30	68.86	66.03	0.0262
Diabetes ^3^	10.82	11.86	9.96	0.0249
Age groups (%)				
20–40 years	33.46	34.92	32.27	0.0045
41–60 years	38.60	38.32	38.83	
61–74 years	20.42	20.52	20.34	
>74 years	7.52	6.24	8.56	
Commune size (%)				
<8.000 inhabitants	35.20	33.83	36.32	0.0849
8.000–40.000 inhabitants	30.67	30.70	30.64	
>40.000 inhabitants	34.13	35.47	33.04	
Marital status (%)				
married	66.71	70.19	63.87	<0.0001
single ^4^	33.29	29.81	36.13	
Level of education ^5^ (%)				
under middle	17.12	14.74	19.06	<0.0001
middle	38.89	36.89	40.52	
academic	19.85	17.09	22.09	
vocational	24.14	31.28	18.33	
Smoking status (%)				
current smokers	23.28	28.95	18.66	<0.0001
past smokers	25.46	33.62	18.82	
never smokers	51.26	37.43	62.52	
Leisure-time physical activity ^6^ (%)				
low level	54.25	54.98	53.67	0.2856
middle level	15.29	14.81	15.68	
high level	28.08	27.50	28.54	
seasonally	2.38	2.71	2.11	
BMI (kg/m^2^) (%)				
underweight (BMI < 18.5)	1.61	0.90	2.20	<0.0001
normal (BMI 18.5–24.99)	34.91	30.07	38.88	
overweight (BMI 25–29.99)	37.25	43.93	31.76	
obesity (BMI ≥ 30)	26.23	25.10	27.16	
Use of phytosterol-enriched margarines (%)	1.90	1.96	1.85	0.7660

* *p* calculated for differences between men and women. ^1^ Hypertension: systolic blood pressure SBP ≥140 mmHg or diastolic blood pressure DBP ≥90 mmHg, or use of antihypertensive drugs. ^2^ Hypercholesterolemia: TC ≥5 mmol/L or LDL-C ≥3 mmol/L or the participant was taking lipid-lowering medication. ^3^ Diabetes: blood glucose level was ≥7.0 mmol/L or diabetes was declared in an interview. ^4^ Singles: widows/widowers, unmarried, divorced, in separation. ^5^ Education level: under middle—no education, partial or completed education for primary level, partial secondary education; middle—secondary education, partial academic education; academic—tertiary education; vocational—vocational based on primary or on middle school. ^6^ Physical activity at leisure (for example, jogging, cycling, swimming, gardening for at least 30 min a day): low level—no such physical activity, once a week or less; middle level—every second or third day; high level—everyday, almost every day; seasonally (e.g., skiing in winter or on the plot in summer).

**Table 2:** Contributions of food categories and individual food products to total plant sterol intake (PS), listed according to diminishing order of contribution.

Food Categories		All N = 5690	Men N = 2554	Women N = 3136	*p* *
Cereals	mg/day (mean ± SD),	90.65 ± 56.38	112.51 ± 63.28	72.85 ± 42.42	<0.0001
	median (IQR)	79.15 (53.94–114.87)	102.44 (69.27–143.02)	66.77 (45.90–91.39)	
	Contribution to PS (%)	32.04	35.08	28.88	<0.0001
	Major sources (% contribution) **	wheat bread (16.65), rolls (6.64), rye bread (5.38)	wheat bread (20.59), rolls (6.85), rye bread (4.82)	wheat bread (12.56), rolls (6.43), rye bread (5.96)	-
Fats	mg/day (mean ± SD),	81.94 ± 92.30	98.34 ± 107.10	68.58 ± 75.63	<0.0001
	median (IQR)	51.65 (19.05–114.67)	64.75 (24.30–138.68)	44.47 (15.76–97.22)	
	Contribution to PS (%)	28.95	30.66	27.20	0.0042
	Major sources (% contribution) **	oils (19.11) *including:* *canola oil* *(16.92),* *sunflower oil (2.06),* *olive* *oil (0.04),* soft margarines (8.33), mayonnaise (1.05)	oils (20.02) *including:* *canola oil* *(18.03),* *sunflower oil (1.88),* *olive* *oil (0.06),* soft margarines (9.08), mayonnaise (1.05)	oils (18.17) *including:* *canola oil* *(15.77),* *sunflower oil (2.25),* *olive* *oil (0.03),* soft margarines (7.56), mayonnaise (1.05)	-
Fruits	mg/day (mean ± SD),	27.76 ± 31.23	25.69 ± 31.46	29.44 ± 30.95	<0.0001
	median (IQR)	20.19 (0–40.38)	17.50 (0–39.37)	21.62 (3.62–42.39)	
	Contribution to PS (%)	9.81	8.01	11.67	<0.0001
	Major sources (% contribution) **	raw fruits (9.78) *including:* *apples (4.47), bananas (1.04), grapes (0.78), pears (0.52), plums (0.48), strawberries (0.37)*	raw fruits (7.98) *including:* *apples (4.10), bananas (0.87), grapes (0.57), pears (0.44), plums (0.40), strawberries (0.27)*	raw fruits (11.65) *including:* *apples (5.42), bananas (1.23), grapes (1.00), pears (0.60), plums (0.55), strawberries (0.48)*	-
Vegetables	mg/day (mean ± SD),	25.37 ± 24.22	26.04 ± 24.16	24.83± 24.26	0.0028
	median (IQR)	20.05 (10.12–33.45)	21.10 (10.53–34.73)	19.22 (9.92–32.34)	
	Contribution to PS (%)	8.97	8.12	9.85	0.0224
	Major sources (% contribution) **	raw vegetables (7.27), *including:* *tomatoes (1.11), carrots (0.90), cabbage (0.84), cauliflowers (0.77), peppers (0.45), beetroot (0.49), lettuce (0.47), cucumbers (0.42),* vegetable preserves (1.32)	raw vegetables (6.45) *including:* *tomatoes (1.02), carrots (0.77), cabbage (0.77), cauliflowers (0.64), beetroot (0.50), peppers (0.39), lettuce (0.38), cucumbers (0.38),* vegetable preserves (1.37)	raw vegetables (8.11) *including:* *tomatoes (1.21), carrots (1.03), cabbage (0.91), cauliflowers (0.91), peppers (0.52), beetroot (0.49), lettuce (0.47), cucumbers (0.42),* vegetable preserves (1.26)	-
Coffee	mg/day (mean ± SD),	19.24 ± 20.39	17.56 ± 20.84	20.61 ± 19.91	<0.0001
	median (IQR)	21.47 (0–26.84)	21.47 (0–26.84)	21.47 (0–26.84)	
	Contribution to PS (%)	6.80	5.48	8.17	<0.0001
Cookies,	mg/day (mean ± SD),	11.57± 23.84	11.36 ± 24.40	11.72 ± 23.40	0.0055
cakes	median (IQR)	0 (0–16.50)	0 (0–15.00)	0 (0–17.60)	
	Contribution to PS (%)	4.08	3.54	4.65	0.0332
Chocolate	mg/day (mean ± SD),	6.46 ± 22.86	6.78 ± 7.20	6.19 ± 21.39	0.0477
products	median (IQR)	0 (0–0)	0 (0–0)	0 (0–0)	
	Contribution to PS (%)	2.28	2.11	2.46	0.3936
Potatoes	mg/day (mean ± SD),	6.12 ± 6.34	7.30 ± 7.20	5.16 ± 5.35	<0.0001
	median (IQR)	6.05 (0–11.01)	6.05 (0–12.10)	4.15 (0–8.07)	
	Contribution to PS (%)	2.16	2.27	2.05	0.5511
Legumes	mg/day (mean ± SD),	3.84 ± 18.56	4.70 ± 21.97	3.13 ± 15.19	0.9949
	median (IQR)	0 (0–0)	0 (0–0)	0 (0–0)	
	Contribution to PS (%)	1.36	1.47	1.24	0.4277
Other food products	mg/day (mean ± SD),	10.02	10.49	9.68	-
	Contribution to PS (%)	3.55	3.26	3.83	0.2434
Total plant sterol intake	mg/day (mean ± SD), median (IQR)	282.97 ± 144.50 255.96 (184.98–347.98)	320.77 ± 160.93 291.76 (209.96–399.07)	252.19 ± 121.20 230.61 (167.73–308.2)	<0.0001
	Contribution to PS (%)	100	100	100	-

* *p* calculated for differences between men and women. ** In the total and each food category, only individual food products with the strongest impact on the total plant sterol intakes were listed.

**Table 3:** Contributions of food categories and individual food products to β-sitosterol intake (β-SIT), listed according to diminishing order of contribution.

Food Categories		All N = 5690	Men N = 2554	Women N = 3136	*p* *
Cereals	mg/day (mean ± SD),	51.37 ± 31.69	63.57 ± 35.62	41.44 ± 23.84	<0.0001
	median (IQR)	44.98 (30.27–64.94)	58.45 (39.20–81.10)	37.87 (26.28–51.81)	
	Contribution to β-SIT (%)	29.19	32.13	26.20	<0.0001
	Major sources (% contribution) **	wheat bread (14.88), rolls (6.04), rye bread (4.75)	wheat bread (18.56), rolls (6.29), rye bread (4.28)	wheat bread (11.14), rolls (5.79), rye bread (5.22)	-
Fats	mg/day (mean ± SD),	50.78 ± 54.93	60.81 ± 63.51	42.61 ± 45.17	<0.0001
	median (IQR)	33.86 (12.70–71.60)	42.15 (16.50–88.00)	28.40 (11.00–61.14)	
	Contribution to β-SIT (%)	28.86	30.73	26.95	0.0017
	Major sources (% contribution) **	oils (18.43) *including:* *canola oil* *(15.88),* *sunflower oil (2.40),* *olive* *oil (0.07), soybean oil (0.07),* soft margarines (9.02), mayonnaise (0.87)	oils (19.40) *including:* *canola oil* *(17.06),* *sunflower oil (2.20),* *olive* *oil (0.09), soybean oil (0.05),* soft margarines (9.87), mayonnaise (0.88)	oils (17.44) *including:* *canola oil* *(14.68),* *sunflower oil (2.60),* *olive* *oil (0.05), soybean oil (0.09),* soft margarines (8.16), mayonnaise (0.86)	-
Fruits	mg/day (mean ± SD),	25.00 ± 27.70	23.21 ± 27.96	26.45 ± 27.41	<0.0001
	median (IQR)	19.50 (0–38.13)	15.90 (0–36.36)	19.50 (3.29–39.00)	
	Contribution to β-SIT (%)	14.20	11.73	16.73	<0.0001
	Major sources (% contribution) **	raw fruits (14.17) *including:* *apples (7.37), bananas (1.29), grapes (1.05), pears (0.81), plums (0.62), strawberries (0.55)*	raw fruits (11.68) *including:* *apples (6.41), bananas (1.08), grapes (0.77), pears (0.69), plums (0.53), strawberries (0.40)*	raw fruits (16.70) *including:* *apples (8.34), bananas (1.51), grapes (1.33), pears (0.93), plums (0.72), strawberries (0.70), peaches (0,56)*	-
Vegetables	mg/day (mean ± SD),	15.31 ± 14.51	15.68 ± 14.69	15.01 ± 14.36	0.0037
	median (IQR)	11.96 (5.88–20.15)	12.60 (6.16–20.84)	11.56 (5.73–19.60)	
	Contribution to β-SIT (%)	8.70	7.92	9.49	0.0347
	Major sources (% contribution) **	raw vegetables (7.11), *including:* *cabbage (0.98), carrots (0.99), tomatoes (0.91), cauliflowers (0.81), peppers (0.54), beetroot (0.43), onion (0.36), cucumbers (0.33),* vegetable preserves (1.23)	raw vegetables (6.36) *including:* *cabbage (0.92), carrots (0.86), tomatoes (0.84), cauliflowers (0.68), peppers (0.47), beetroot (0.43), onion (0.38), cucumbers (0.32),* vegetable preserves (1.28)	raw vegetables (7.88) *including:* *cabbage (1.06), carrots (1.13), tomatoes (0.99), cauliflowers (0.94), peppers (0.61), beetroot (0.42), onion (0.34), cucumbers (0.35),* vegetable preserves (1.18)	-
Coffee	mg/day (mean ± SD),	9.91 ± 10.50	9.05 ± 10.74	10.62 ± 10.26	<0.0001
	median (IQR)	11.06 (0–13.83)	11.06 (0–13.83)	11.06 (0–13.83)	
	Contribution to β-SIT (%)	5.64	4.57	6.72	0.0005
Cookies,	mg/day (mean ± SD),	7.04 ± 14.04	6.97 ± 14.69	7.10 ± 13.49	0.0058
cakes	median (IQR)	0 (0–10.20)	0 (0–10.00)	0 (0–10.40)	
	Contribution to β-SIT (%)	4.00	3.52	4.49	0.0645
Chocolate	mg/day (mean ± SD),	3.89 ± 13.77	4.10 ± 14.76	3.73 ± 12.89	0.0485
products	median (IQR)	0 (0–0)	0 (0–0)	0 (0–0)	
	Contribution to β-SIT (%)	2.22	2.07	2.36	0.4699
Potatoes	mg/day (mean ± SD),	4.35 ± 4.50	5.18 ± 5.12	3.67 ± 3.80	<0.0001
	median (IQR)	4.30 (0–7.82)	4.30 (0–8.60)	2.95 (0–5.73)	
	Contribution to β-SIT (%)	2.47	2.62	2.32	0.4742
Legumes	mg/day (mean ± SD),	2.21 ± 11.91	2.71 ± 14.03	1.80 ± 9.83	0.9852
	median (IQR)	0 (0–0)	0 (0–0)	0 (0–0)	
	Contribution to β-SIT (%)	1.25	1.37	1.14	0.4522
Other food products	mg/day (mean ± SD),	6.12	6.61	5.71	-
	Contribution to β-SIT (%)	3.47	3.34	3.60	0.5732
Total β-sitosterol intake	mg/day (mean ± SD), median (IQR)	175.98 ± 88.00 160.85 (115.80–218.15)	197.89 ± 98.28 180.84 (131.20–246.86)	158.14 ± 74.02 146.28 (105.89–196.13)	<0.0001
	Contribution to β-SIT (%)	100	100	100	-

* *p* calculated for differences between men and women. ** In the total and each food category, only individual food products with the strongest impact on the total plant sterol intakes were listed.

**Table 4:** Contributions of food categories and individual food products to campesterol intake (CAMP), listed according to diminishing order of contribution.

Food Categories		All N = 5690	Men N = 2554	Women N = 3136	*p* *
Fats	mg/day (mean ± SD),	26.55 ± 35.53	32.02 ± 41.34	22.10 ± 29.23	<0.0001
	median (IQR)	12.52 (3.75–37.85)	16.70 (4.65–45.65)	10.10 (3.00–31.11)	
	Contribution to CAMP (%)	44.95	46.30	43.47	0.0311
	Major sources (% contribution) **	oils (34.60) *including:* *canola oil* *(33.43),* *sunflower oil (1.07), soybean oil (0.08),* *olive* *oil (0.01),* soft margarines (8.11), mayonnaise (1.87)	oils (35.50) *including:* *canola oil* *(34.48),* *sunflower oil (0.94), soybean oil (0.06),* *olive* *oil (0.01),* soft margarines (8.58), mayonnaise (1.81)	oils (33.61) *including:* *canola oil* *(32.26),* *sunflower oil (1.21), soybean oil (0.11),* *olive* *oil (0.01),* soft margarines (7.60), mayonnaise (1.94)	-
Cereals	mg/day (mean ± SD),	18.79 ± 11.57	23.34 ± 13.00	15.09 ± 8.65	<0.0001
	median (IQR)	16.50 (11.11–23.84)	21.46 (14.48–30.02)	13.79 (9.37–19.06)	
	Contribution to CAMP (%)	31.81	33.74	29.67	0.0009
	Major sources (% contribution) **	wheat bread (16.30), rolls (6.91), rye bread (5.49)	wheat bread (19.52), rolls (6.92), rye bread (4.76)	wheat bread (12.74), rolls (6.91), rye bread (6.29)	-
Vegetables	mg/day (mean ± SD),	3.04 ± 3.81	3.06 ± 3.90	3.04 ± 3.75	0.2297
	median (IQR)	2.00 (0.72–3.99)	2.09 (0.74–4.08)	1.95 (0.69–3.91)	
	Contribution to CAMP (%)	5.16	4.43	5.97	0.0098
	Major sources (% contribution) **	fresh vegetables (4.29) *including:* *cauliflowers (1.07), cabbage (0.93), carrots (0.70), peppers (0.51), tomatoes (0.36),* vegetable preserves (0.68)	fresh vegetables (3.62) *including:* *cabbage (0.75), cauliflowers (0.71), carrots (0.49), peppers (0.36), tomatoes (0.28),* vegetable preserves (0.67)	fresh vegetables (5.04) *including:* *cauliflowers (1.07), cabbage (0.93), carrots (0.70), peppers (0.51), tomatoes (0.36),* vegetable preserves (0.69)	-
Cookies,	mg/day (mean ± SD),	2.99 ± 6.30	2.99 ± 6.69	3.00 ± 5.95	0.0049
cakes	median (IQR)	0 (0–3.90)	0 (0–3.60)	0 (0–4.16)	
	Contribution to CAMP (%)	5.07	4.32	5.90	0.0071
Coffee	mg/day (mean ± SD),	3.15 ± 3.33	2.87 ± 3.41	3.37 ± 3.26	<0.0001
	median (IQR)	3.51 (0–4.39)	3.51 (0–4.39)	3.51 (0–4.39)	
	Contribution to CAMP (%)	5.33	4.15	6.63	<0.0001
Fruits	mg/day (mean ± SD),	1.52 ± 2.47	1.38 ± 2.53	1.63 ± 2.42	<0.0001
	median (IQR)	0.60 (0–1.91)	0.54 (0–1.64)	0.72 (0.15–2.23)	
	Contribution to CAMP (%)	2.57	1.99	3.21	0.0044
	Major sources (% contribution) **	raw fruits (2.56) *including:* *apples (0.61), bananas (0.53), grapes (0.30), mandarins (0.27), plums (0.19), oranges (0.17)*	raw fruits (1.98) *including:* *apples (0.51), bananas (0.42), grapes (0.22*), *mandarins (0.21),* *plums (0.16), oranges (0.11)*	raw fruits (3.20) *including:* *apples (0.72), bananas (0.64), grapes (0.40), mandarins (0.34), oranges (0.24), plums (0.23)*	-
Chocolate	mg/day (mean ± SD),	0.68 ± 2.41	0.71 ± 2.59	0.65 ± 2.25	0.0462
products	median (IQR)	0 (0–0)	0 (0–0)	0 (0–0)	
	Contribution to CAMP (%)	1.15	1.03	1.28	0.3669
Potatoes	mg/day (mean ± SD),	0.37 ± 0.38	0.44 ± 0.44	0.31 ± 0.32	<0.0001
	median (IQR)	0.37 (0–0.67)	0.37 (0–0.73)	0.25 (0–0.49)	
	Contribution to CAMP (%)	0.63	0.64	0.61	0.9213
Legumes	mg/day (mean ± SD),	0.35 ± 1.75	0.42 ± 2.06	0.28 ± 1.44	0.9950
	median (IQR)	0 (0–0)	0 (0–0)	0 (0–0)	
	Contribution to CAMP (%)	0.59	0.61	0.55	0.6768
Other food products	mg/day (mean ± SD),	1.62	1.93	1.36	-
	Contribution to CAMP (%)	2.74	2.79	2.67	0.8152
Total campesterol intake	mg/day (mean ± SD), median (IQR)	59.06 ± 41.44 47.45 (31.53–74.39)	69.16 ± 47.68 56.71 (37.17–86.39)	50.83 ± 33.38 40.88 (27.80–64.90)	<0.0001
	Contribution to CAMP (%)	100	100	100	-

* *p* calculated for differences between men and women. ** In the total and each food category, only individual food products with the strongest impact on the total plant sterol intakes were listed.

**Table 5:** Contributions of food categories and individual food products to stigmasterol intake (STIG), listed according to diminishing order of contribution.

Food Categories		All N = 5690	Men N = 2554	Women N = 3136	*p* *
Coffee	mg/day (mean ± SD),	6.18 ± 6.55	5.64 ± 6.70	6.62 ± 6.40	<0.0001
	median (IQR)	6.90 (0–8.63)	6.90 (0–8.63)	6.90 (0–8.63)	
	Contribution to STIG (%)	25.10	21.41	28.53	<0.0001
Vegetables	mg/day (mean ± SD),	5.72 ± 5.48	6.00 ± 5.63	5.49 ± 5.34	0.0007
	median (IQR)	4.34 (1.90–7.96)	4.55 (1.95–8.48)	4.12 (1.85–7.45)	
	Contribution to STIG (%)	23.22	22.76	23.66	0.4179
	Major sources (% contribution) **	raw vegetables (17.90) *including:* *tomatoes (4.63), beets (1.90), cucumbers (1.82), carrots (1.80), parsley (1.88), green beans (1.88), lettuce (1.24), celery (1.08),* vegetable preserves (4.10)	raw vegetables (17.15) *including:* *tomatoes (4.48), beets (2.02), cucumbers (1.83), carrots (1.64), parsley (1.89), green beans (1.52), lettuce (1.17), celery (1.12),* vegetable preserves (4.62)	raw vegetables (18.60) *including:* *tomatoes (4.76), beets (1.79), cucumbers (1.81), carrots (1.96), parsley (1.88), green beans (2.21), lettuce (1.31), celery (1.04),* vegetable preserves (3.62)	-
Fats	mg/day (mean ± SD),	4.15 ± 4.93	5.02 ± 5.79	3.44 ± 3.97	<0.0001
	median (IQR)	2.59 (0.81–5.77)	3.23 (1.05–7.10)	2.28 (0.66–4.97)	
	Contribution to STIG (%)	16.85	19.05	14.84	<0.0001
	Major sources (% contribution) **	soft margarines (11.82), oils (3.75), mixed fats (0.65), mayonnaise (0.60)	soft margarines (13.90), oils (3.69), mixed fats (0.79), mayonnaise (0.63)	soft margarines (9.91), oils (3.80), mixed fats (0.52), mayonnaise (0.56)	-
Cereals	mg/day (mean ± SD),	3.19 ± 2.26	3.91 ± 2.43	2.60 ± 1.93	<0.0001
	median (IQR)	2.72 (1.82–3.98)	3.53 (2.36–5.02)	2.33 (1.57–3.19)	
	Contribution to STIG (%)	12.93	14.83	11.18	<0.0001
	Major sources (% contribution) **	wheat bread (6.48), rolls (2.57), rye bread (2.48), cereals (0,66)	wheat bread (8.49), rolls (2.69), rye bread (2.44), cereals (0,47)	wheat bread (4.62), rolls (2.29), rye bread (2.69), cereals (0,84)	-
Chocolate	mg/day (mean ± SD),	1.58 ± 5.59	1.66 ± 6.00	1.51 ± 5.22	0.0474
products	median (IQR)	0 (0–0)	0 (0–0)	0 (0–0)	
	Contribution to STIG (%)	6.40	6.29	6.52	0.7579
Legumes	mg/day (mean ± SD),	1.21 ± 6.04	1.47 ± 7.23	0.99 ± 4.86	0.9904
	median (IQR)	0 (0–0)	0 (0–0)	0 (0–0)	
	Contribution to STIG (%)	4.91	5.58	4.28	0.0208
Fruits	mg/day (mean ± SD),	0.92 ± 1.81	0.82 ± 1.71	1.00 ± 1.88	<0.0001
	median (IQR)	0.18 (0–0.88)	0.15 (0–0.60)	0.22 (0.02–1.13)	
	Contribution to STIG (%)	3.73	3.13	4.29	0.0211
	Major sources (% contribution) **	raw fruits (3.72) *including:* *bananas (1.68), apples (0.40), nectarines (0.30*), *plums (0.29), peaches (0.40)*	raw fruits (3.12) *including:* *bananas (1.33), apples (0.37), nectarines (0.27*), *plums (0.26), peaches (0.25)*	raw fruits (4.28) *including:* *bananas (1.68), apples (0.44), nectarines (0.33*), *plums (0.32), peaches (0.53)*	-
Potatoes	mg/day (mean ± SD),	0.61 ± 0.63	0.73 ± 0.72	0.52 ± 0.53	<0.0001
	median (IQR)	0.60 (0–1.10)	0.60 (0–1.21)	0.41 (0–0.81)	
	Contribution to STIG (%)	2.48	2.77	2.22	0.1861
Cookies,	mg/day (mean ± SD),	0.59 ± 1.52	0.57 ± 1.62	0.61 ± 1.44	0.0004
cakes	median (IQR)	0 (0–0.40)	0 (0–0.25)	0 (0–0.50)	
	Contribution to STIG (%)	2.40	2.17	2.61	0.2589
Other food products	mg/day (mean ± SD),	0.48	0.54	0.44	-
	Contribution to STIG (%)	1.98	2.01	1.88	0.7530
Total stigmasterol intake	mg/day (mean ± SD), median (IQR)	24.63 ± 14.49 22.10 (14.53–30.92)	26.36 ± 16.02 23.49 (15.14–32.91)	23.22 ± 12.94 21.11 (14.16–29.19)	<0.0001
	Contribution to STIG (%)	100	100	100	-

* *p* calculated for differences between men and women. ** In the total and each food category, only individual food products with the strongest impact on the total plant sterol intakes were listed.

**Table 6:** Comparison of total and individual sterol intakes (in mg and in mg/1000 kcal) by men and women.

Plant Sterols (mg)	Men	Women	*p*-Value
Total plant sterols	320.8	252.2	<0.0001
Total plant sterols/1000 kcal	141.0	154.2	<0.0001
β-sitosterol	197.8	158.1	<0.0001
β-sitosterol/1000 kcal	87.1	96.8	<0.0001
Campesterol	69.2	50.8	<0.0001
Campesterol/1000 kcal	29.8	30.4	0.2279
Stigmasterol	26.4	23.2	<0.0001
Stigmasterol/1000 kcal	12.0	14.8	<0.0001

## Data Availability

All data in this study are made available upon request to the authors at the following e-mail address: anna.witkowska@umb.edu.pl or awaskiewicz@ikard.pl.

## Supplementary Materials

The following are available online at https://www.mdpi.com/article/10.3390/nu13082722/s1, Table S1: Content of selected plant sterols (stigmasterol, campesterol, beta-sitosterol) and total plant sterols in food products (mg/100 g of product), Table S2: Content of selected plant sterols (stigmasterol, campesterol, beta-sitosterol) and total plant sterols in dishes (mg/100 g of dish).
