# Supplementary material for: Assessment of Plant Sterols in the Diet of Adult Polish Population with the Use of a Newly Developed Database
Source: Nutrients. 2021 Aug 7;13(8):2722. doi: 10.3390/nu13082722 (PMC8398305; doi:10.3390/nu13082722)
Supplement: Supplementary file 1 [file nutrients-13-02722-s001.zip › nutrients-1258194-Supplementary Table S1.pdf]

Table S1: Content of selected plant sterols (stigmasterol, campesterol, beta-sitosterol) and total plant sterols in food products (mg/100g of product) *).

No.	Food products	Code	Stigmasterol mg/100g	Campesterol mg/100g	Beta-sitosterol mg/100g	Plant sterols total mg/100g	Retention factor
1	Yogurt with bananas, 1,5% fat	131002	0.09	0.075	0.55	0.715	1
2	Yogurt with bilberries, 1,5% fat	131003	0	0.05	1.11	1.32	1
3	Yogurt with apricots, 1,5% fat	131004	0.02	0.06	0.58	0.67	1
4	Yogurt with strawberries, 1,5% fat	131005	0.01	0.015	0.545	0.59	1
5	Yogurt with cherries, 1,5% fat	131006	0	0.01	0.55	0.565	1
6	Fresh cheese dessert, with cocoa	142001	0.628	0.246	1.552	2.52	1
7	Fresh cheese dessert, with strawberries	142002	0.02	0.03	1.09	1.18	1
8	Cheese fresh, with strawberries, homogenized	142009	0.02	0.03	1.09	1.18	1
9	Ice cream, fruit	151001	0.02	0.03	1.09	1.18	1
10	Milk dessert, with rice and apples	161001	0.21	0.216	2.13	2.64	1
11	Fish-vegetable salad "Paprykarz szczeciński", canned	431001	2.547	10.202	17.66	33.29	1
12	Cod salad, canned	431004	2.147	9.842	16	30.61	1
13	Makerel salad, canned	431005	2.147	9.842	16	30.61	1
14	Sardine in tomato sauce, canned	431007	0.85	0.14	1.2	2.35	1
15	Herring in tomato sauce, canned	431010	0.85	0.14	1.2	2.35	1
16	Oil, safflower	511001	23	36	168	274	1
17	Oil, corn	511002	55.3	153	538	746.3	1
18	Oil, palm	511003	8.4	15.7	38.5	62.6	1
19	Oil, rapeseed, low erucic acid content	511004	2.38	260	368	630.38	1
20	Oil, rapeseed "Uniwersalny"	511005	2.38	260	368	630.38	1
21	Oil, rapeseed, cold pressed	511006	2.38	260	368	630.38	1
22	Oil, rapeseed and soybean	511007	28.89	159	260.5	448.4	1
23	Oil, sesame	511008	86.89	90.3	322.76	499.95	1
24	Oil, sunflower	511009	34.5	31.5	210	290	1
25	Oil, soybean	511010	55.4	58	153	266.4	1
26	Oil, wheatgerm	511011	0	122	370	525	1
27	Oil, olive	511012	1.02	5.68	126	132.7	1
28	"Planta", vegetable fat	511013	25	147	243	425	1
29	Oil, grapeseed	511014	15	13	98	126	1

30	Margarine, hard, 80% fat	512015	35	75	275	385	1
31	Margarine, soft, 80% fat	512016	35	75	275	385	1
32	Margarine, soft, 70% fat	512017	25	31	81	137	1
33	Margarine, soft, 60% fat	512018	25	28	74	127	1
34	Margarine, soft, 50% fat	512019	25	28	74	127	1
35	Margarine, soft, 45% fat	512020	25	28	74	127	1
36	Butter, "Extra"	521001	0	0	4	4	1
37	Butter, ordinary	521002	0	0	4	4	1
38	Mixed fat, vegetable and animal, 60% fat	531004	25	28	74	127	1
39	Wheat flour, type 500	611001	0.4	6.5	21	31.8	0.62
40	Wheat flour, type 550	611003	0.4	6.5	21	31.8	0.62
41	Wheat, grain	611014	0.8	5.1	17.4	32	0.62
42	Buckwheat groats	621001	2.6	11	81	94.6	0.62
43	Millet groats	621002	3.9	9.9	39	55	0.62
44	Pearl flour	621003	1	1	6	9.7	0.62
45	Wheat grits	621005	0.8	5.1	17.4	32	0.62
46	Rice white polished	621006	2	1.8	8.3	13.4	0.62
47	Pasta made without eggs	631001	0.2	0.5	4.6	7.1	0.62
48	Rye bread from whole grain	641001	2.9	17	47	86	1
49	Rye bread	641002	2.2	11	29	51	1
50	Rye bread "Pytlowy"	641004	2.2	11	28	51	1
51	Rye brown bread	641005	2.2	11	28	51	1
52	Rye brown bread Litewski"	641006	2.2	11	28	51	1
53	Rye brown bread with honey	641007	2.2	11	28	51	1
54	Rye brown bread "Wytrawny"	641008	2.2	11	28	51	1
55	Rye brown bread with soybeans and sunflower seeds	641009	2.2	11	28	51	1
56	Rye bread "Sitkowy"	641010	2.2	11	28	51	1
57	Rye bread "Staropolski"	641012	2.2	11	28	51	1
58	Rye bread "Wileński"	641013	2.2	11	28	51	1
59	Rye bread "Pumpernickel"	641014	2.9	17	47	86	1
60	Rye bread with dried plums	641015	2.2	11	28	51	1
61	Rye bread, extruted	641016	2.2	11	28	51	1
62	Wheat-rye bread "Baltonowski"	642001	1.8	11	30	54	1
63	Crispbread	642002	2.9	17	47	86	1
64	Wheat-rye bread "Mazowiecki"	642003	1.8	11	30	54	1

65	Wheat-rye bread with sunflower seeds	642004	1.8	11	30	54	1
66	Wheat-rye bread with soya beans	642005	1.8	11	30	54	1
67	Wheat-rye bread with milk	642006	1.8	11	30	54	1
68	Wheat-rye bread "Poleski"	642008	2.2	11	28	51	1
69	Wheat-rye bread "Wiejski"	642010	1.8	11	30	54	1
70	Wheat-rye bread "Zwykły"	642013	1.8	11	30	54	1
71	Wheat-rye bread "Beskidzki"	642014	2.2	11	28	51	1
72	Wheat-rye bread with dried fruits mixed	642015	2.2	11	28	51	1
73	Wheat-rye bread with rolled oat	642016	2.2	11	28	51	1
74	Wheat-rye bread with grains	642017	2.2	11	28	51	1
75	Wheat rolls with onion	642020	2.2	11	28	51	1
76	Rolls, French	643001	0.9	12	31	50	1
77	Rolls, graham	643002	2.2	11	28	51	1
78	Wheat rolls	643003	1.8	11	30	54	1
79	Bread crumbs	643004	1.2	16	35	56	1
80	Wheat bread, graham	643005	2.2	11	28	51	1
81	Wheat bread	643006	1.8	11	30	54	1
82	Wheat bread with wheat grains	643007	1.8	11	30	54	1
83	Wheat bread, extruded	643008	1.8	11	30	54	1
84	Wheat rolls for hod dog	644001	1.8	11	30	54	1
85	Wheat rolls with milk	644002	0.6	6.8	18	29	1
86	Wheat rolls with rolled oats	644003	1.8	11	30	54	1
87	Wheat rolls with soya flour	644004	1.8	11	30	54	1
88	Wheat rolls "Wrocławskie"	644005	1.8	11	30	54	1
89	Wheat rolls "Kajzerki"	644006	1.8	11	30	54	1
90	Wheat toasted bread	644007	1.8	11	30	54	1
91	Wheat rolls "Strucla Wrocławska"	644008	1.8	11	30	54	1
92	Wheat-graham toasted bread	644009	1.8	11	30	54	1
93	Wheat rolls "Ciabata"	644010	1.8	11	30	54	1
94	Wheat rolls with whey	644011	1.8	11	30	54	1
95	Wheat rolls "Szwed"	644013	1.8	11	30	54	1
96	Sweet yeast buns	645001	0.6	6.8	18	29	1
97	Sweet yeast buns "Chałki"	645002	0.6	6.8	18	29	1
98	Semi-puff pastry with jam	645003	0.6	6.8	18	29	1
99	Rolls, crescent	645005	0.6	6.8	18	29	1

100	Rolls yeast with fresh cheese	645006	0.6	6.8	18	29	1
101	Rolls yeast with poppy seeds	645007	0.6	6.8	18	29	1
102	Rolls yeast with plum jam	645008	0.6	6.8	18	29	1
103	Corn crisps	651001	21	51	148	276	1
104	Muesli with dried fruit	651002	0.9	3.8	21.3	29.7	1
105	Muesli with raisins and nuts	651003	6.5	6.4	21.3	40.7	1
106	Wheat, bran	651004	5.1	20.2	56.6	115.4	1
107	Barley flakes	651005	1	1	6	9.7	0.62
108	Corn flakes	651006	1	4.3	17	26	1
109	Corn flakes fortified with vitamins and iron	651007	1	4.3	17	26	1
110	Rolled oats	651008	1.6	5.5	32	39.1	0.62
111	Wheat flakes	651009	1	1	6	9.7	0.62
112	Rye flakes	651010	1	1	6	9.7	0.62
113	Rice puffed, roasted	651011	0.6	0.1	2	2.8	1
114	Wheat, germs	651012	3.2	94	230	344	1
115	Corn flakes with sugar, fortified with vitamins and iron	651013	0.3	0.2	4	5.8	1
116	Corn flakes with honey and nuts, fortified with vitamins and iron	651014	0.3	0.2	4	5.8	1
117	Pastry with meat	661001	0.6	6.8	18	29	1
118	Pizza with meat and herbs	661002	0	7.3	14.9	23.2	1
119	Pizza with mushrooms and onion	661003	0	7.3	14.9	23.2	1
120	Aubergine	711001	0.6	0.2	2	2.9	2.36
121	Beets greens	711002	0	0	0	21	0.59
122	Broad beans	711003	8.1	10.2	24.4	145	0.95
123	Broccoli, green	711004	1.1	6.9	31	39	0.57
124	Brussels sprouts	711005	0.38	8	34	43	0.61
125	Beetroot	711006	5.7	0.6	9.1	17.1	0.59
126	Onion	711007	0.57	0.82	7	8.4	0.53
127	Horseradish	711008	0.26	3.3	14	17.56	0.59
128	Squash-summer	711009	8.4	0.4	7.3	16.7	0.65
129	Chicory	711010	7.9	3.3	16.7	30.9	0.87
130	Garlic, raw	711011	0.5	2	8.7	11.2	0.53
131	Pumpkin	711012	8.4	0.4	7.3	16.7	0.42
132	Beans, white, dried	711013	86.2	15.2	85.1	186.5	0.54
133	String-beans, raw	711015	11.12	1.96	10.98	24.06	0.95
134	Peas, yellow, dried	711016	26	36.4	269.1	349.05	0.54

135	Peas, green, raw	711017	4	5.6	41.4	53.7	0.48
136	Cauliflower	711019	3.7	9.5	26	40	0.63
137	Kohlrabi	711020	0.2	2.8	9.4	13	0.59
138	Cabbage, white	711021	0.2	2.8	9.4	13	0.57
139	Cabbage, red	711022	0.2	2.8	9.4	13	0.57
140	Cabbage, Shantung	711023	0.03	1.6	6.8	8.5	0.57
141	Dill	711025	13.3	1.6	15.5	32.5	1
142	Sweetcorn	711026	0.4	9.1	34.1	43.6	1
143	Carrot	711027	2.8	2.2	11	16	0.59
144	Cucumber	711028	2.9	0.2	3.8	7.3	1.01
145	Pepper, red	711029	0.2	4.2	16.4	22	1.01
146	Pepper, green	711030	0.33	2	4.9	7.23	0.91
147	Parsley, root	711032	11.5	1.2	13.6	28.8	0.59
148	Parsley, leaves	711033	13.3	1.6	15.5	32.5	0.87
149	Tomato	711034	1.7	0.28	2.4	4.7	0.88
150	Leek	711035	0.06	0.61	7.3	8.1	0.32
151	Turnip	711037	0.26	3.3	14	17.56	0.62
152	Radishes	711038	0	0	4.4	9	0.59
153	Lettuce	711039	7.9	3.3	16.7	30.9	0.87
154	Celeriac	711040	8.6	2.7	8.9	20.2	0.59
155	Celery	711041	7	2.7	8.9	20	1
156	Lentils red, dried	711042	4	6	47	57	0.54
157	Lentil sprouts, raw	711043	2	3	23.5	28.5	1
158	Soya bean, dried	711044	16.3	20.94	64.98	161	0.54
159	Soya bean sprouts, raw	711045	5.4	1.8	7.5	15.2	1
160	Chives	711047	0	5.1	16.2	22	0.87
161	Asparagus, raw	711048	3.7	9.5	26	40	1.31
162	Spinach	711049	2.9	0.8	5.4	10.6	0.87
163	Potato	711052	0.38	0.23	2.7	3.8	0.69
164	Peas green, frozen	721005	4	5.6	41.4	53.7	0.95
165	Carrot, frozen	721007	2.8	2.2	11	16	1
166	Tomato, frozen	721009	1.7	0.28	2.4	4.7	0.88
167	Spinach, frozen	721010	2.9	0.8	5.4	10.6	0.87
168	Sweetcorn, frozen	721019	0.4	9.1	34.1	43.6	1
169	Soup, beetroot and vegetables, frozen	722001	5.7	0.6	9.1	17.1	1

170	Spring vegetables, frozen	722002	5.524	5.798	20.814	32.591	1
171	Vegetables with pasta, frozen	722003	2.862	3.149	12.707	19.845	1
172	Vegetables with rice, frozen	722004	3.762	3.799	14.557	22.995	1
173	Vegetable salad "Paprykarz", frozen	722007	3.7005	1.087	6.568	11.816	1
174	Vegetable salad with sweetcorn, frozen	722008	4.21	4.21	21.75	30.74	1
175	Mexican salad, frozen	722009	4.21	4.21	21.75	30.74	1
176	Scandinavian salad, frozen	722010	3.7005	1.087	6.568	11.816	1
177	Vegetables-mix, frozen	722011	5.716	1.791	11.16	19.45	1
178	Soup "Wintry", frozen	722016	5.524	5.798	20.814	32.591	1
179	Vegetable salad, frozen	722022	4.21	4.21	21.75	30.74	1
180	Vegetable with chicken and rice, frozen	722023	2.8096	2.8592	10.8156	17.0564	1
181	Vegetables with spices, frozen	722024	3.7005	1.087	6.568	11.816	1
182	Vegetables with chicken and pasta, frozen	722026	2.2696	2.4692	9.7056	15.1664	1
183	Vegetables with potatoes and ham cooked and smoked, frozen	722028	2.3236	2.3882	9.1356	14.1764	1
184	Vegetables with pasta and ham cooked and smoked, frozen	722029	2.2696	2.4692	9.7056	15.1664	1
185	String-beans, drained, canned	731001	11.12	1.96	10.98	24.06	0.95
186	Beans, white, canned	731002	11.12	1.96	10.98	24.06	0.95
187	Peas, green, drained, canned	731003	4	5.6	41.4	53.7	0.95
188	Sauerkraut	731004	0.09	3.2	11	15	0.41
189	Tomato, paste, canned	731005	8.5	1.4	12	23.5	1
190	Soya flour, whole fat	731006	16.3	20.94	64.98	161	0.54
191	Cucumber, pickled with salt and dill	731007	2.9	0.2	3.8	7.3	1.01
192	pepper red, pickled, canned	731008	0.33	2	4.9	7.23	1
193	Tomato, ketchup	731010	8.5	1.4	12	23.5	1
194	Sweetcorn, canned	731012	0.4	9.1	34.1	43.6	1
195	Polish dill pickles, canned	731013	2.9	0.2	3.8	7.3	1
196	Polish dill pickles with red pepper, canned	731014	2.9	0.2	3.8	7.3	1
197	Polish dill pickles "Pikle", canned	731015	2.9	0.2	3.8	7.3	1
198	Onion pickled, canned	731016	0.57	0.82	7	8.4	1
199	Swedish cucumbers, canned	731018	3.7005	1.087	6.568	11.816	1
200	Mushroom, common, row	741001	0	2	0	2	1
201	Mushroom common pickled, canned	743001	0	2	0	2	1
202	Gooseberries	811001	0.2	0.2	5.6	6	1.01
203	Pineapple	811002	0.44	3.8	11	17	1.01
204	Water-melon	811003	0.26	0.18	0.91	1.35	1.01

205	Avocado	811004	2	5	76	83	1.01
206	Banana	811005	1.8	1.5	11	14.3	1.01
207	Peach	811006	1.8	0.58	13	15.38	1.01
208	Lemon	811007	1.3	3.3	13	18	1.01
209	Bilberries	811008	0	1	22.2	26.4	1.01
210	Sweet cherries	811009	0	0.2	11	11.3	1.01
211	Grapefruit	811010	1	2.5	15	18.5	1.01
212	Pears	811011	0	0.27	12	12.27	1.01
213	Guava	811012	1.5	2.7	19.4	24.4	1.01
214	Apple	811013	0.1	0.36	13	13.46	1.01
215	Kiwi fruit	811014	1.4	0.44	7.2	9.1	1.01
216	Raspberries	811015	0	0.9	23.3	27.4	1.01
217	Mandarin orange	811016	0.78	4	12	16.78	1.01
218	Mango	811017	1.5	2.7	19.4	24.4	1.01
219	Melon	811018	0.49	0.17	1.2	1.86	1.01
220	Apricots	811019	0.4	1.2	11.6	13.4	1.01
221	Nectarine	811020	1.8	0.58	13	15.38	1.01
222	Orange	811022	1	3	20	24	1.01
223	Whitecurrants	811023	0.2	0.2	5.6	6	1.01
224	Blackcurrants	811024	0	0.5	8.1	8.8	1.01
225	Redcurrants	811025	0.2	0.2	5.6	6	1.01
226	Wild strawberries	811026	0.2	0.3	10.9	11.8	1.01
227	Plums	811027	0.7	1.1	10.6	13	1.01
228	Strawberries	811028	0.2	0.3	10.9	11.8	1.01
229	Grapes	811029	0.2	1.2	12.3	14.8	1.01
230	Sour cherries	811030	0	0.2	11	11.3	1.01
231	Raspberries, frozen	821002	0	0.9	23.3	27.4	1
232	Plums without stones, frozen	821004	0.7	1.1	10.6	13	1.01
233	Strawberries, frozen	821005	0.2	0.3	10.9	11.8	1
234	Sour cherries without stones, frozen	821006	0	0.2	11	11.3	1.01
235	Fruit-mix, frozen	822001	0.2	0.3	10.9	11.8	1
236	Banans, dried	831001	7.03	5.86	42.97	55.86	1
237	Dates, dried	831002	5.43	8.53	82.17	100.76	1
238	Figs, dried	831003	5.43	8.53	82.17	100.76	1
239	Apples, dried	831004	0.76	2.73	98.48	101.97	1

240	Apricots, dried	831005	2.94	8.82	85.29	98.53	1
241	Raisins, dried	831006	1.07	6.42	65.78	79.14	1
242	Plums with stones, dried	831007	5.43	8.53	82.17	100.76	1
243	Pineapple jam, low-sugar	841001	0	0	0	12	1
244	Peach jam, low-sugar	841002	0	0	0	12	1
245	Blackcurrants jam, low-sugar	841003	0	0	0	12	1
246	Blackcurrants jam, high-sugar	841004	0	0	0	12	1
247	Bilberries jam, low-sugar	841005	0	0	0	12	1
248	Apricot jam, low-sugar	841007	0	0	0	12	1
249	Orange jam, low-sugar	841008	0	0	0	12	1
250	Plums jam, low-sugar	841009	0	0	0	12	1
251	Plums jam, high-sugar	841010	0	0	0	12	1
252	Strawberries jam, low-sugar	841011	0	0	0	12	1
253	Strawberries jam, high-sugar	841012	0	0	0	12	1
254	Sour cherries jam, low-sugar	841013	0	0	0	12	1
255	Sour cherries jam, high-sugar	841014	0	0	0	12	1
256	Plums jam, "Powidła śliwkowe"	841015	0	0	0	12	1
257	Strawberries jam, low-sugar, fortified vitamin C	841016	0	0	0	12	1
258	Pineapple in sirup, canned	842001	0.44	3.8	11	17	1
259	Olives green, in brine, canned	842002	0.29	1.1	34	35.39	1
260	Peach in sirup, canned	842003	1.8	0.58	13	15.38	1
261	Strawberries sirup	842004	0	0	0	12	1
262	Almonds, sweet	911001	3	9	118	130	1
263	Peanuts	911002	13	15	76	116	1
264	Coconut	911003	15	5	33	53	1
265	Hazelnuts	911005	1	7	108	116	1
266	Pistachio nuts, dried	911006	5	10	198	214	1
267	Walnuts	911007	0	5	87	92	1
268	Poppy seeds, dried	1011001	7	29	109	145	1
269	Sesame seeds	1011002	22	53	232	714	1
270	Sunflower seeds	1011003	15.8	20.9	140.4	222.5	1
271	Pumpkin seeds	1011004	0	3	13	16	1
272	Linen seeds	1011005	11	45	90	213	1
273	Cocoa, powder	1131001	31.4	12.3	77.6	126	1
274	Cocoa drink, powder, fortified vitamins	1131002	31.4	12.3	77.6	126	1

275	"Bounty Milk", bar	1141001	23	10	56	94	1
276	"Mars", bar	1141002	23	10	56	94	1
277	"Milky Way", bar	1141003	23	10	56	94	1
278	"Snickers", bar	1141004	23	10	56	94	1
279	"Twix", bar	1141005	23	10	56	94	1
280	Chocolate, plain	1141006	23	10	56	94	1
281	Chocolate, bitter	1141007	31	12	86	129	1
282	Chocolate, milk	1141009	23	10	56	94	1
283	Chocolate, milk with hazelnuts	1141010	23	10	56	94	1
284	Chocolate, filled	1141011	23	10	56	94	1
285	Chocolate, cream with hazeltunts	1141012	23	10	56	94	1
286	Chocolate, white	1141013	23	10	56	94	1
287	Sponge cakes with fruit jam, chocolate-coated	1151001	4.3	13	34	55	1
288	Biscuits	1151002	4.3	13	34	55	1
289	Biscuits "Corso"	1151003	4.3	13	34	55	1
290	Biscuits sugar-coated	1151004	4.3	13	34	55	1
291	Biscuits with chocolate	1151005	4.3	13	34	55	1
292	Biscuits, chocolate-coated	1151006	4.3	13	34	55	1
293	Biscuits with peanuts, chocolate-coated	1151007	4.3	13	34	55	1
294	Biscuits with cocoa	1151008	4.3	13	34	55	1
295	Biscuits with coconut	1151009	4.3	13	34	55	1
296	Biscuits with chocolate filling	1151010	4.3	13	34	55	1
297	Biscuits with fruit jam filling	1151011	4.3	13	34	55	1
298	Biscuits with sesame seeds	1151012	6.07	17	53.8	120.9	1
299	Biscuits with milk filling	1151013	4.3	13	34	55	1
300	Sticks with caraway seeds	1151014	4.3	13	34	55	1
301	Gingerbread with fruit jam filling, chocolate-coated	1151015	5.9	19	41	71	1
302	Gingerbread with fruit jam filling, glazed	1151016	5.9	19	41	71	1
303	Sticks slated	1151017	2	8.2	23	36	1
304	Wafers with filling	1151018	4	7.2	26	44	1
305	Wafers with filling, chocolate-coated	1151019	4	7.2	26	44	1
306	Cakes "Sezamki"with sesame seeds	1151020	22	53	232	714	1
307	Halva with vanilla	1152001	22	53	232	714	1
308	Halva with cocoa	1152002	22	53	232	714	1
309	Sponge cakes with fruit jam, chocolate-coated	1161001	0	9.4	20	29.4	1

310	Rolls yeast with apples	1161002	0.5	6.5	16.4	26.5	1
311	Yeast cake	1161003	0.5	6.5	16.4	26.5	1
312	Short-cake	1161004	2.6	20	36	61	1
313	Puff-pastry cakes with plums	1161005	2.6	20	36	61	1
314	Cakes "Karpatka"	1161006	0	9.4	20	29.4	1
315	Cakes "Napoleonka"	1161007	0	9.4	20	29.4	1
316	Layer cakes with fresh fruit	1161008	0	9.4	20	29.4	1
317	Eclairs	1161009	0	9.4	20	29.4	1
318	Cakes yeast with bilberries	1161010	0.5	6.5	16.4	26.5	1
319	Cake with dried fruits mixed	1161011	5.9	19	41	71	1
320	Gingerbread with dried fruits mixed	1161012	5.9	19	41	71	1
321	Yeast cake with crust pastry	1161013	2.7	6.6	19	30	1
322	Cream puffs	1161014	0	9.4	20	29.4	1
323	Rolled dough with poppy seeds	1161015	2.7	6.6	19	30	1
324	Puff-pastry crescent with apple	1161016	2.6	20	36	61	1
325	Layer cake with pineapple	1161021	0	9.4	20	29.4	1
326	Cakes with cream	1161022	2.6	20	36	61	1
327	Yeast cake "Luksusowa"	1161023	1.7	16.9	21.3	44.4	1
328	Roll yeast with apple and crust pastry	1161025	2	6	18	27	1
329	Yeast cake with strawberries	1161026	2.7	6.6	19	30	1
330	Cake "Ponczowe" with cream	1161027	2.6	20	36	61	1
331	Chrust	1161028	2.6	20	36	61	1
332	Cake from fresh aplles	1161029	2	6	18	27	1
333	Cake puff "Groszek ptysiowy"	1161031	2.3	7.8	18.7	32.9	1
334	Doughnut	1161032	0.5	6.5	16.4	26.5	1
335	Layer cake "Wuzetka"	1161035	0	9.4	20	29.4	1
336	Tea, infusion without sugar	1211001	0.0011	0.0092	0.08	0.0903	1
337	Coffee, infusion, without sugar	1211002	3.45	1.755	5.53	10.735	1
338	Pineapple juice	1212001	0.023	0.071	0.42	0.514	1
339	Lemon juice	1212003	0.023	0.071	0.42	0.514	1
340	Grapefruit juice	1212004	0.023	0.071	0.42	0.514	1
341	Apple juice	1212005	0.0026	0.027	0.21	0.2396	1
342	Carrot juice	1212006	1.27	0.677	2.7	4.647	1
343	Carrot and peach juice	1212007	0.224	0.607	2.5	3.331	1
344	Carrot and apple juice	1212008	0.224	0.607	2.5	3.331	1

345	Carrot and apple and orange juice	1212009	0.224	0.607	2.5	3.331	1
346	Carrot and orange juice	1212010	0.224	0.607	2.5	3.331	1
347	Carrot, apple and tropical fruits juice	1212011	0.224	0.607	2.5	3.331	1
348	Orange juice	1212012	0.023	0.071	0.42	0.514	1
349	Tomato juice	1212013	0.331	0.155	0.36	0.846	1
350	Vegetable juice	1212014	0.596	0.242	0.74	1.578	1
351	Carrot and banana and apple juice	1212015	0.224	0.607	2.5	3.331	1
352	Carrot, peach and apple juice	1212016	0.224	0.607	2.5	3.331	1
353	Carrot, apricot and apple juice	1212017	0.224	0.607	2.5	3.331	1
354	Apple and mango juice	1212019	0.0026	0.027	0.21	0.2396	1
355	Grapefruit juice with aspartame	1212020	0.023	0.071	0.42	0.514	1
356	Orange and grapefruit juice	1212022	0.03	0.139	2.1	2.269	1
357	Multifruit juice from polish fruits	1212023	0.0026	0.027	0.21	0.2396	1
358	Peach and orange juice	1212026	0.023	0.071	0.42	0.514	1
359	Grape white juice	1212027	0.023	0.071	0.42	0.514	1
360	Beetroot juice "Barszczyk czerwony"	1212028	0.04	0.047	0.42	0.507	1
361	Multifruit juice from tropical fruits	1212029	0.023	0.071	0.42	0.514	1
362	Coca Cola, Pepsi Cola	1213001	0.0013	0.008	0.05	0.0593	1
363	Fruit juice drink, carbonated ready to drink	1213002	0.0008	0.0058	0.06	0.0666	1
364	Lemonade	1213003	0.019	0.073	0.48	0.572	1
365	Apple drink	1213004	0.0026	0.027	0.21	0.2396	1
366	Apple and sour cherry drink	1213005	0.0026	0.027	0.21	0.2396	1
367	Apple and grape red drink	1213006	0.0026	0.027	0.21	0.2396	1
368	Apple and mentha drink	1213007	0.0026	0.027	0.21	0.2396	1
369	Orange drink	1213008	0.023	0.071	0.42	0.514	1
370	Grape white drink	1213009	0.023	0.071	0.42	0.514	1
371	Pineapple nectar	1214001	0.023	0.071	0.42	0.514	1
372	Peach nectar	1214002	0.023	0.071	0.42	0.514	1
373	Blackcurrant nectar	1214003	0.023	0.071	0.42	0.514	1
374	Sour cherry nectar	1214006	0.023	0.071	0.42	0.514	1
375	Carrot, banana and apple juice, fortified vitamin E nad C	1215001	0.224	0.607	2.5	3.331	1
376	Multifruit juice from exotic fruits, fortified vitamins	1215009	0.023	0.071	0.42	0.514	1
377	Blackcurrant nectar, fortified vitamins	1217003	0.03	0.139	2.1	2.269	1
378	Sour cheery nectar, fortified vitamins	1217006	0.03	0.139	2.1	2.269	1
379	Beer	1221001	0.005	0.0293	0.235	0.2693	1

380	Champagne	1221002	0	0	0.094	0.094	1
381	Vermouth, sweet	1221003	0	0	0.094	0.094	1
382	Wine, white, medium	1221004	0	0	0.094	0.094	1
383	Wine white sweet	1221005	0	0	0.094	0.094	1
384	Wine, white, dry	1221006	0	0	0.094	0.094	1
385	Wine, red	1221007	0	0	0.094	0.094	1
386	Potato crisps, bacon flavour	1411001	14	15	45	88	1
387	Potato crisps with paprika	1411002	14	15	45	88	1
388	Potato crisps, salted	1411003	14	15	45	88	1
389	Mayonnaise, home-made, from repeseed oil	1421002	24	181	251	484	1
390	Mayonnaise, home-made, from sunflower oil	1421003	24	181	251	484	1
391	Mustard	1421004	2.5	26.5	74.4	103.4	1
392	Soya beans goulash	2091901	8.15	2.55	32.49	80.5	0.54
393	Soya beans, cutletes	2091902	8.15	2.55	32.49	80.5	0.54
394	Soya beans, cutletes (without eggs)	2091903	8.15	2.55	32.49	80.5	0.54
395	Soya beans paste with tomato paste	2091904	8.15	2.55	32.49	80.5	1
396	Soya beans, paste, pâté	2091905	8.15	2.55	32.49	80.5	1
397	Soya beans, boiled	2091906	8.15	2.55	32.49	80.5	0.54

*) Database compatible with "Tables of composition and nutritional value of food" - Poland, 2011
