# Supplementary material for: Assessment of Plant Sterols in the Diet of Adult Polish Population with the Use of a Newly Developed Database
Source: Nutrients. 2021 Aug 7;13(8):2722. doi: 10.3390/nu13082722 (PMC8398305; doi:10.3390/nu13082722)
Supplement: Supplementary file 1 [file nutrients-13-02722-s001.zip › nutrients-1258194-Supplementary Table S2.pdf]

Table S2: Content of selected plant sterols (stigmasterol, campesterol, beta-sitosterol) and total plant sterols in dishes (mg/100g of dish) *).

No.	Dishes	Code	Stigmasterol mg/100g	Campesterol mg/100g	Beta-sitosterol mg/100g	Plant sterols, total mg/100g
1	Soup, beetroot, creamed	2011001	0.8523	0.1618	1.5838	2.873
2	Soup, beetroot and vegetables with string-beans	2011002	3.0599	1.0656	5.718	10.3622
3	Soup, beetroot and vegetables with white beans	2011003	3.2998	2.2113	9.3369	15.0308
4	Soup, beet greens	2011004	0.2073	0.0659	0.4894	2.086
5	Soup, sauerkraut	2011005	0.011	0.2614	0.8438	1.1842
6	Soup, cabbage	2011006	0.8287	1.2379	4.7582	6.9763
7	Soup, cream of green pea	2011007	1.107	1.6234	11.6661	15.1887
8	Soup, "Krupnik" with buckwheat groats	2011008	0.9767	1.2166	6.2515	8.6067
9	Soup, "Krupnik" with pearl barley groats	2011009	0.9371	0.9686	4.3915	6.5012
10	Soup, "Krupnik" with rice	2011011	0.9619	0.9885	4.4486	6.593
11	Soup, onion	2011012	0.7929	2.0985	7.348	10.3785
12	Soup, lemon with rice	2011013	1.6134	2.7246	10.4488	14.9532
13	Soup, white bean	2011014	7.9156	1.4357	7.9424	17.3179
14	Soup, yellow pea	2011015	2.2464	3.145	23.2502	30.1579
15	Soup, mushroom, creamed	2011016	0.0085	0.229	0.2044	0.4662
16	Soup, vegetable	2011017	3.4781	1.4368	6.7829	11.8191
17	Soup, vegetable, creamed	2011018	1.5622	1.6143	6.3016	9.7007
18	Soup, cauliflower	2011019	1.2014	1.946	6.0937	9.5225
19	Soup, dill-pickled cucumber	2011021	0.7188	0.8549	3.7856	5.5186
20	Soup, apple	2011022	0.0434	0.1563	5.6459	5.8457
21	Soup, tomato	2011023	0.8262	0.2038	1.4325	2.5903
22	Soup, tomato with pasta	2011024	0.6135	0.841	3.3144	4.8412
23	Soup, tomato with rice	2011025	0.6507	0.8745	3.4688	5.0904
24	Soup, tomato, creamed	2011026	0.8262	0.2038	1.4325	2.5903
25	Soup, celeriac	2011028	1.4223	1.1422	4.3216	6.9814
26	Soup, celeriac with croutons	2011029	2.8049	3.3038	11.2227	18.6273
27	Soup, sorrel	2011030	0.705	1.5806	5.7604	8.0943
28	Soup, spinach with potatoes	2011031	0.8779	0.3385	2.2867	3.8928
29	Soup, string bean	2011032	5.144	1.057	6.3691	12.8034
30	Soup, leek with pasta	2011033	1.1271	1.0881	5.0662	7.3729

31	Soup, green pea	2011034	1.9077	2.2214	13.3625	18.2084
32	Soup, potato	2011035	0.949	0.966	4.5036	6.6277
33	Soup, potato, creamed	2011036	0.4626	0.857	3.6627	5.1484
34	Soup, "Żurek", sour rye	2011037	0.2019	0.1622	1.4345	2.0589
35	Soup, "Neapolitan" with Emmental cheese	2011038	0.7843	1.0725	4.0589	6.0807
36	Soup, wheat grits with vegetable broth	2011039	0.5741	0.2763	1.3125	2.4416
37	Soup, beetroot	2011901	1.6013	0.2578	2.7495	5.0226
38	Soup, chicken with pasta	2011906	0.4622	0.1922	1.0393	1.7316
39	Soup, dill-pickled cucumber, creamed	2011912	0.7018	0.2373	1.7549	2.8884
40	Soup, milk with wheat grits	2012002	0.0397	0.253	0.863	1.5872
41	Soup, milk with noodles dropped	2012003	0.0099	0.1612	0.5208	0.7886
42	Soup, milk with rolled oats	2012005	0.0893	0.3069	1.7856	2.1818
43	Soup, milk with rice	2012006	0.0992	0.0893	0.4117	0.6646
44	Cocoa with milk, powder	2012901	1.256	0.492	3.104	5.04
45	Cod fillets in vegetables, boiled	2021001	3.9335	5.8367	22.466	32.4109
46	Cod fillets with vegetables and tomatoes, braised	2021002	2.0473	31.8535	47.5772	81.7252
47	Cod fillets, breaded and fried	2021003	0.5757	44.2418	64.9112	110.4438
48	Carp with vegetables, galantine	2021004	1.5407	0.4798	2.7532	4.9852
49	Carp, boiled	2021005	1.4116	0.4233	2.3709	4.3472
50	Cod chops minced, fried	2021006	0.7164	51.5948	75.4168	128.9284
51	Cod balls minced, in vegetables, boiled	2021007	0.8827	1.7964	5.3616	9.151
52	Cod fillets, grilled	2021008	0.4423	13.2612	19.5888	33.3571
53	Salmon, saute, fried	2021009	0.3719	39.2418	55.9812	95.74
54	Salmon, grilled	2021010	0.4423	13.2612	19.5888	33.3571
55	Veal, boiled in sauce	2031001	2.4713	3.504	13.3587	19.5339
56	Veal chops in sauce, braised	2031002	0.568	28.71	43.5	74.538
57	Veal cutlets, breaded and fried	2031003	0.6094	42.4015	62.851	106.7428
58	Veal, braised	2031004	0.3016	26.1326	37.3664	63.8491
59	Veal, tripe, dressed	2031901	0.704	0.2431	1.2626	2.3256
60	Chicken minced chops, fried	2032001	0.5386	21.08	33.01	56.6866
61	Hen, gelantine	2032002	0.7342	0.2319	1.2943	2.3343
62	Chicken with vegetables, boiled	2032003	4.6773	3.9057	15.9145	24.6655
63	Chicken, roasted	2032004	2.2157	4.5139	16.6349	23.3828
64	Chicken, boiled with sauce	2032005	1.0703	0.536	2.6993	4.522
65	Chicken fillets, saute, fried	2032006	0.0124	0.2015	0.651	0.9858

66	Chicken fillets, breaded, fried	2032007	0.132	1.76	3.85	6.16
67	Turkey cutlets, fried	2032008	0.0124	0.2015	0.651	0.9858
68	Pork steaks, braised	2033001	1.0599	2.4112	8.7708	12.3386
69	Pork steaks with vegetables, braised	2033002	2.1384	2.8141	10.6079	15.7027
70	Pig's knuckles, gelantine	2033003	0.5117	0.1715	0.9144	1.6419
71	Pork cutlets, breaded and fried	2033004	0.6044	46.9224	68.8516	117.1419
72	Pork, braised	2033006	0.1517	13.0838	18.9012	32.1614
73	Pork roll, roasted	2033007	0.342	2.09	5.7	10.26
74	Pork loin, roasted	2033009	0.1583	13.0565	18.8823	32.0978
75	Pork steaks, fried	2033010	0.249	26.0893	37.1346	63.5214
76	Pig's liver, saute, fried	2033011	0.3034	26.1675	37.8024	64.3227
77	Pork, boiled	2033012	1.7855	0.6007	3.5366	6.1322
78	Pork ribs, braised	2033013	0.1567	13.1644	19.1616	32.5557
79	Pork, knuckle, boiled	2033901	0.5076	0.168	0.9015	1.6236
80	Pork ribs, braised	2033909	0.0364	0.1442	0.6965	0.9381
81	Beef steaks, chopped, fried	2034001	0.2084	18.2968	26.3725	44.9025
82	Beef steaks, braised	2034002	1.0599	2.4112	8.7708	12.3386
83	Beef steaks with vegetables, braised	2034003	3.3627	3.2501	13.6506	20.6268
84	Beef Stroganoff, braised	2034004	0.3155	21.0187	30.4518	51.8992
85	Beef filled, fried	2034005	0.243	26.0806	37.0604	63.4323
86	Beef goulash, braised	2034006	0.4089	21.0219	30.6403	52.1738
87	Beef filled, roasted	2034007	3.15	6.75	24.75	34.65
88	Beef, braised	2034008	0.2129	20.9426	30.0161	51.2445
89	Beef, boiled	2034010	1.2446	0.6694	3.3036	5.3892
90	Beef roulade with pork fat, braised	2034011	0.0401	0.2047	0.8918	1.2338
91	Beef and pork minced chops, braised	2035001	0.587	23.2363	36.3878	62.5724
92	Beef and pork minced cutlets, fried	2035002	0.5498	33.3948	49.6568	84.8018
93	Pate from mixed meat, baked	2035003	0.2253	1.1652	3.5565	6.0678
94	Beef and pork minced, roasted	2035004	1.76	5.72	17.9	27.4
95	Beef and pork minced balls, boiled	2035005	1.6581	2.7428	8.8447	15.7471
96	Sauerkraut with sausage and meat, "Bigos", stewed	2041001	0.8911	7.6702	16.3068	25.12
97	Beans baked with meat in tomato sauce	2041002	13.5685	5.8833	25.72	45.2282
98	Cabbage leaves stuffed with meat and rice in tomato sauce	2041003	0.5756	3.788	8.1872	12.8939
99	Pepper bell stuffed with meat and rice	2041004	0.681	2.8807	11.7436	16.2178
100	Risotto with meat and vegetables	2041005	1.1932	0.5747	3.0061	5.0626

101	Cucumbers sliced in sour cream	2051001	3.19	0.22	4.18	8.03
102	Lettuce with tomato, cucumber and oil, salad	2051002	7.845	3.699	22.38	36.64
103	Lettuce with oil, salad	2051003	13.096	7.692	45.503	71.381
104	Lettuce with sour cream, salad	2051004	7.9	3.3	16.7	30.9
105	Chicory and apple, salad	2051005	12.555	8.4	54.41	80.285
106	White cabbage, salad	2051006	2.0305	4.778	21.89	30.06
107	Sauerkraut, salad	2051007	1.8745	4.147	22.7	29.955
108	Carrot, salad	2051008	2.27	1.868	12.7	16.838
109	Carrot and apple, salad	2051009	3.61	2.9884	20.99	27.5884
110	Cucumber and tomato, salad	2051010	2.8305	0.353	4.69	8.275
111	Fruit, salad	2051011	0.399	0.8712	15.702	17.2402
112	Friut and vegetables, slad	2051012	8.775	6.2078	41.388	59.758
113	Pepper bell, sald	2051013	4.333	4.854	27.56	38.69
114	Tomato, salad	2051014	1.7684	0.3784	3.24	5.708
115	Leek and apple, salad	2051015	4.59	4.9655	40.325	51.863
116	Radish, salad	2051016	0	0.51	3.82	6.7
117	Celeric and apple with oil, salad	2051017	13.105	5.85	30.73	50.805
118	Celeric, apple and carrot, salad	2051018	5.324	2.436	21.08	28.84
119	Shantung cabbage with oil, salad	2051019	0.2145	12.7936	27.893	41.1031
120	Shantung cabbage with mayonnaise, salad	2051020	0.3359	23.7138	44.462	68.5992
121	Vegetable mixed with yogurt, salad	2051021	1.4355	1.756	7.645	11.3275
122	Brussels sprouts, boiled	2052001	0.3142	6.336	25.938	33.156
123	Beetroot, boiled	2052002	5.7208	4.208	20.2156	31.3522
124	Beets with apples, cooked	2052003	5.1465	3.4732	18.5341	28.2914
125	Beets, cooked with roux	2052004	3.7163	0.4874	6.3147	11.6703
126	Beets in sour cream, cooked	2052005	3.7043	0.47	6.1663	11.4922
127	Beets cooked, grated with horseradish	2052006	4.0479	0.5806	7.1036	12.9356
128	Pumpkin with tomato	2052007	4.5875	0.3287	4.4396	9.7774
129	String beans, boiled with butter	2052008	13.7692	2.9006	14.6103	31.3941
130	Peas, green, boiled, with butter	2052009	1.92	2.688	19.872	25.776
131	Cauliflower, boiled, with butter	2052010	2.8332	7.662	20.706	31.92
132	Cabbage, white, stewed, with roux	2052011	0.131	1.694	5.7668	7.9824
133	Carrot and green peas, boiled	2052012	3.8029	5.4166	25.6848	35.4671
134	Carrot, boiled, with roux	2052014	1.9874	1.6382	8.0484	11.7223
135	Vegetable salad, cooked, with mayonnaise	2052015	12.0714	6.868	45.0925	66.7512

136	Green peas, salad	2052016	7.5413	7.5805	53.6552	72.1359
137	Red cabbage, salad	2052017	2.5788	3.8556	23.4436	31.1834
138	Spinach, boiled, with roux	2052018	3.7919	1.1649	7.4376	14.4245
139	Vegetable salad, cooked, with mayonnaise and yogurt	2052019	8.2614	19.298	32.3925	62.9512
140	Mushrooms, fried	2053001	0	3	0	3
141	Potato pancakes with yeast, fried	2061001	0.5289	2.0049	6.6836	11.4087
142	Meat and pasta pudding	2061002	0.7428	1.1813	4.3779	6.4966
143	Buckwheat groats, boiled with vegetable fat	2061003	1.3448	4.228	25.588	31.1608
144	Buckwheat groats, boiled with lard	2061004	0.6448	2.728	20.088	23.4608
145	Pearl barley groats, boiled, with vegetable fat	2061005	0.917	1.717	6.802	9.8049
146	Pearl barley groats, boiled, with lard	2061006	0.217	0.217	1.302	2.1049
147	Noodles, spoon-dropped, boiled	2061007	0.124	2.015	6.51	9.858
148	Noodles, batter, boiled	2061008	0.0546	0.8866	2.8644	4.3375
149	Dumplings with plums, boiled	2061009	0.4409	1.2406	7.0748	9.555
150	Potato dumplings, boiled	2061010	0.3267	1.2065	5.2482	7.7482
151	Potato dumplings, bolied, with butter	2061011	0.3188	1.2017	5.1923	7.6695
152	Yeast cake with filling " Kulebiak"	2061012	0.2336	19.2881	29.2754	49.4499
153	Dumplings with fresh cheese and potato, bolied	2061013	0.1237	1.0787	3.4348	5.2195
154	Dumplings with fresh cheese, boiled	2061014	2.8488	6.723	24.002	33.8916
155	Pasta boiled, with fresh cheese and butter	2061017	0.062	1.0075	3.255	4.929
156	Pancakes filled with cabbage, fried	2061018	0.1689	2.0952	7.1702	10.0774
157	Pancakes filled with fresh cheese, fried	2061019	0.0446	0.7254	2.3436	3.5489
158	Dumplings with potato filling "Ruskie", boiled	2061020	0.2138	1.177	4.5587	6.7916
159	Polish dumplings with meat, boiled	2061022	0.1247	6.2292	10.8005	17.7592
160	Polish dumplings with fresh cheese, boiled	2061024	0.062	1.0075	3.255	4.929
161	Polish dumplings with strawberries, boiled	2061025	0.2423	2.386	11.2205	15.0322
162	Risotto with meat	2061026	0.7458	16.1799	24.6135	41.8675
163	Rice, boiled with vegetable fat	2061027	1.196	1.9464	7.5584	11.0232
164	Rice boiled, with lard	2061028	0.434	0.3906	1.8011	2.9078
165	Rice with apples, baked	2061029	1.0414	2.1091	14.3349	17.7007
166	Potato, mashed	2061030	0.3671	0.2222	2.6082	3.6708
167	Potato, with vegetable fat	2061031	1.7828	3.2317	13.72	19.2281
168	Potato dumplings	2061032	0.7099	0.4423	5.1225	7.1709
169	Potato dumplins with meat	2061033	0.6143	1.3158	6.6352	9.6461
170	Potato pancakes, fried	2061034	0.7929	39.483	59.0165	99.921

171	Pasta with white cabbage, baked	2061035	0.2378	14.3594	23.1439	38.0255
172	Potato noodles, "Śląskie", boiled	2061036	2.0741	4.2178	16.8411	23.6979
173	Potato cutlets from cooked potatoes, fried	2061037	0.8057	35.1245	53.7075	90.5392
174	Pasta "Łazanki", with white cabbage, baked	2061038	0.1352	1.7553	5.9333	8.5513
175	Pancake batter sponge-cake style, fried	2061039	0.0794	1.2896	4.1664	6.3091
176	Pancake batter, fried	2061040	0.0992	1.612	5.208	7.8864
177	Rice boiled	2061042	0.496	0.4464	2.0584	3.3232
178	French fries	2061902	1.1012	0.6665	7.8246	11.0124
179	Dumplings with white cabbage and mushrooms	2061906	0.6903	2.7916	8.6676	12.8341
180	Yeast pancakes with apples, fried	2061908	1.8107	3.6117	15.7299	22.1823
181	Potato, boiled	2061909	0.4033	0.2441	2.8653	4.0326
182	Fine pasta made with eggs "Zacierka"	2061910	0.0826	1.342	4.3357	6.5654
183	Eggs, scrambled	2071002	0.7	1.5	5.5	7.7
184	Eggs, chopped cutlets, fried	2071003	2.352	6.56	21.6	31.86
185	Omelette biscuit with spinach	2071004	3.0499	1.1979	6.8094	12.8408
186	Omelette biscuit	2071901	0.0496	0.806	2.604	3.9432
187	Omelette, natural	2071902	6.37	13.65	50.05	70.07
188	Pears with vanilla sauce	2081002	0	0.2591	11.514	11.7731
189	Apples, baked	2081003	0.1202	0.4327	15.6247	16.1776
190	Apple pie	2081004	8.21	18.9553	77.1282	105.164
191	Jelly starch with redcurrant	2081005	0.0303	0.1061	2.0756	2.2422
192	Apple compote	2081007	0.0566	0.1721	6.1166	6.3452
193	Sour cherry compote	2081008	0	0.0626	3.4441	3.538
194	Apple sauce	2081010	0.0636	0.2291	8.2719	8.5646
195	Milk drink with strawberries	2081011	0.0929	0.1394	5.0641	5.4823
196	Sponge roll with jam	2081012	0.0347	0.5642	1.8228	6.2402
197	Strawberries in sweet gelatine	2081013	0.0808	0.1212	4.4036	4.7672
198	Strawberries with whipped cream	2081014	0.1232	0.1848	6.7155	7.27
199	Gooseberry and strawberry compote	2081902	0.0707	0.0808	2.5149	2.7068
200	Redcurrant compote	2081903	0.101	0.101	2.828	3.03
201	Apple and redcurrant compote	2081904	0.0564	0.1719	6.1109	6.3392
202	Apple and plum compote	2081906	0.1717	0.3313	6.0802	6.7044
203	Plum compote	2081907	0.3732	0.6055	5.55	6.8377
204	Strawberry compote	2081908	0.0808	0.1212	4.4036	4.7672
205	Steak "Tartar"	2091001	0.7223	15.8171	26.9658	43.5467

206	Eggs, paste	2091002	1.1194	34.208	49.7705	85.2685
207	Eggs and fish smoked, paste	2091003	0.3487	34.1671	49.3089	83.868
208	Fresh cheese and fish smoked, paste	2091004	0.3094	33.8	47.84	81.9494
209	Horseradish sauce	2611001	1.5033	3.6461	13.3162	18.6663
210	Greek sauce	2611002	10.1858	7.3389	24.0381	42.0194
211	Mushroom sauce	2611003	1.8127	4.8973	15.2179	22.0979
212	Meat sauce	2611005	0.5773	0.4536	2.4432	3.6698
213	Mustard sauce	2611006	1.5624	4.0076	13.2709	19.1788
214	Tomato sauce	2611007	2.6174	4.1721	15.8614	22.9801
215	Chives sauce	2611008	2.2236	5.3068	19.1894	26.9395
216	Tartare sauce	2611009	33.12	36.0222	95.8235	165.0522

*) Database compatible with "Tables of composition and nutritional value of food" - Poland, 2011

The content of plant sterols in dishes was calculated on the basis of recipes of these dishes, efficiency, retention factor and the content of plant sterols in products included in the dishes.
